# A role of eosinophils in mediating the anti-tumour effect of cryo-thermal treatment

**DOI:** 10.1038/s41598-019-49734-5

**Published:** 2019-09-13

**Authors:** Shengguo Jia, Wentao Li, Ping Liu, Lisa X. Xu

**Affiliations:** 1 School of Biomedical Engineering and Med-X Research Institute, Shanghai Jiao Tong University, Shanghai, P.R. China; 2 Fudan University Shanghai Cancer Center, Shanghai, China; 3 Department of Oncology, Shanghai Medical College, Fudan University, Shanghai, China

**Keywords:** Cancer immunotherapy, Immunoediting

## Abstract

Previous, we established a novel therapeutic approach to tumour of cryo-thermal therapy, which can induce durable anti-tumour memory immunity mediated by CD4^+^ T cell, and contribute to prolonged survival in B16F10 murine melanoma model and 4T1 murine mammary carcinoma. It has become apparent that innate immune cells are involved in the regulation of adaptive T cell immunity. Our previous studies revealed that cryo-thermal therapy induced M1 macrophage polarization and DCs maturation were required for the shaping of systemic long-lived T cell mediated anti-tumour memory immunity. Eosinophils are multifunctional innate effector cells and there is lack of knowledge on the role of eosinophils in cryo-thermal-induced anti-tumour immunity. This study revealed that cryo-thermal therapy activated eosinophils in spleen at early stage following the treatment. Furthermore, cryo-thermal-activated eosinophils exerted versatile immunologic regulation from innate immunity to anti-tumour adaptive immunity, such as M1 macrophage polarization, DCs maturation, differentiation of CD4-CTL subtypes and enhanced cytotoxicity of CD8^+^ T cells. Our study indicated that the cryo-thermal-activated eosinophils was essential for the shaping of durable anti-tumour memory immunity. Thus, our results present a new concept for eosinophils mediated anti-tumour immunity after cryo-thermal therapy.

## Introduction

Accumulating evidences show that inducing durable anti-tumour immunity are essential in cancer therapy. Local thermal ablation, such as conventional RFA (using temperature >60 °C), or cryosurgery (using temperature −20 °C to −40 °C) is generally accepted for the treatment of metastatic tumour in clinic^1–3^. Either RFA or cryosurgery alone was reported to elicit immunological responses. RFA modulates immune system and induces anti-tumour immunity^4,5^. RFA can induce tumour cell necrosis and subsequent release of tumour antigen^4^. The physical barriers of tumours are ablated after the treatment, which results in increasing infiltration of immune cells, including NK cells, APCs and T cells^4,5^. In addition, cryoablation can also induce potent anti-tumour immune response through recovering tumour-educated dendritic cells (DCs) or activating of effector T cells^6,7^. However, there is no lasting and enough response to establish a sustained anti-tumour immunity for clinical applications^8,9^.

To enhance the curative power of thermal ablation, we establish a new kind of therapeutic approach to tumour of cryo-thermal therapy through the alternative cooling and heating of tumour tissues^10,11^. Cryo-thermal therapy significantly causes tumour cell necrosis and markedly improves cancer survival on 4T1 murine mammary carcinoma and B16F10 murine melanoma model^11–13^. Cryo-thermal therapy induces a specific and durable anti-tumour memory immunity which dependents on antigen-specific CD4^+^ T cells^12^. The modulation and sustain of T cell-mediated adaptive immunity is critical for efficacious tumour cell killing and generating effective anti-tumour memory immunity^13^. The innate immune cells are capable of bridging innate and adaptive anti-tumour immune responses^14^. A large body of data demonstrated that activating the innate immune response could stimulate both local and systemic antitumour T-cell responses. The innate immune cells, such as DCs and macrophages, are capable of cross-prime T cells leading to the activation of adaptive immune response^15^. Infiltration of mature, immunogenic DCs into tumours confers immune activation^16^. Fully mature DCs can present antigen to CD4^+^ and CD8^+^ T cells leading to trigger antigen-specific cytotoxic T lymphocytes (CTLs)^17^. Tumour-associated macrophages are divided into alternative M2 and classical M1 macrophages. M2 macrophages have inherent immune suppressive activity and promote tumour growth^18^. In contrast, M1 macrophages are characterized by high expression level of pro-inflammatory factors and presentation of tumour-specific antigens to T cells^19^. Previously, cryo-thermal-induced M1 macrophage polarization was exclusively responsible for the subsequent DC maturation, differentiation of CD4-CTL and Th1 subsets, which was crucial for mediating long-term anti-tumour memory immunity after cryo-thermal therapy^20^. But whether other innate immune cells also mediated cryo-thermal-induced anti-tumour memory immunity should be addressed.

Eosinophils are multifunctional granulocytes linked to the modulation of diverse inflammatory response including allergic diseases, helminth infections, tissue injury and tumour immunity^21^. Growing evidence supported eosinophils contributing to anti-tumour immunity. Clinically, the infiltration of tumour-associate tissue eosinophils (TATE) are usually indicated a good prognosis, such as head and neck cancer, colorectal cancer, and prostate cancer^22–24^. Eosinophils can exert cytotoxic properties for tumour cell upon activating via the release of several cytotoxic granules, including eosinophil cationic protein (ECP), eosinophil derived neurotoxin (EDN), major basic protein (MBP) and eosinophils peroxidase (EPO). Meanwhile, eosinophils are also a major source of numerous immunologically active factors, for example, Th1 and Th2 cytokines and chemokines^25^. Moreover, eosinophils can also intact with other immune cells such as DCs, macrophages and T cells to modulate anti-tumour immunity^26–28^. However, if eosinophils contribute to anti-tumour immunity, especially, the role of eosinophils in cryo-thermal-induced long-lasting anti-tumour memory immunity remained unclear.

In this study, we explored whether eosinophils would serve as key cells that contributed to tumour-specific T cell responses mediated by cryo-thermal therapy. We investigated the phenotype and function of eosinophils after local cryo-thermal therapy using a murine B16F10 melanoma model. And eosinophils depletion *in vivo* after cryo-thermal therapy was constructed evaluate the role of cryo-thermal-activated eosinophils in shaping of long–term anti-tumour immunity. We discovered that cryo-thermal therapy induced the activation of eosinophils at early stage following the treatment. Cryo-thermal-activated eosinophils play a crucial role in M1 macrophage polarization, DCs maturation, functional differentiation of CD4^+^ T cells, generation of cytotoxic CD8^+^ T cells, and finally triggering long-lasting anti-tumour memory immunity. Thus, our study presented a new concept of eosinophils mediated anti-tumour immunity after cryo-thermal therapy that would lead to novel therapeutic strategies.

## Results

### Cryo-thermal therapy induced an *increased proportion of eosinophils* and activation of eosinophils

**Figure 1:**
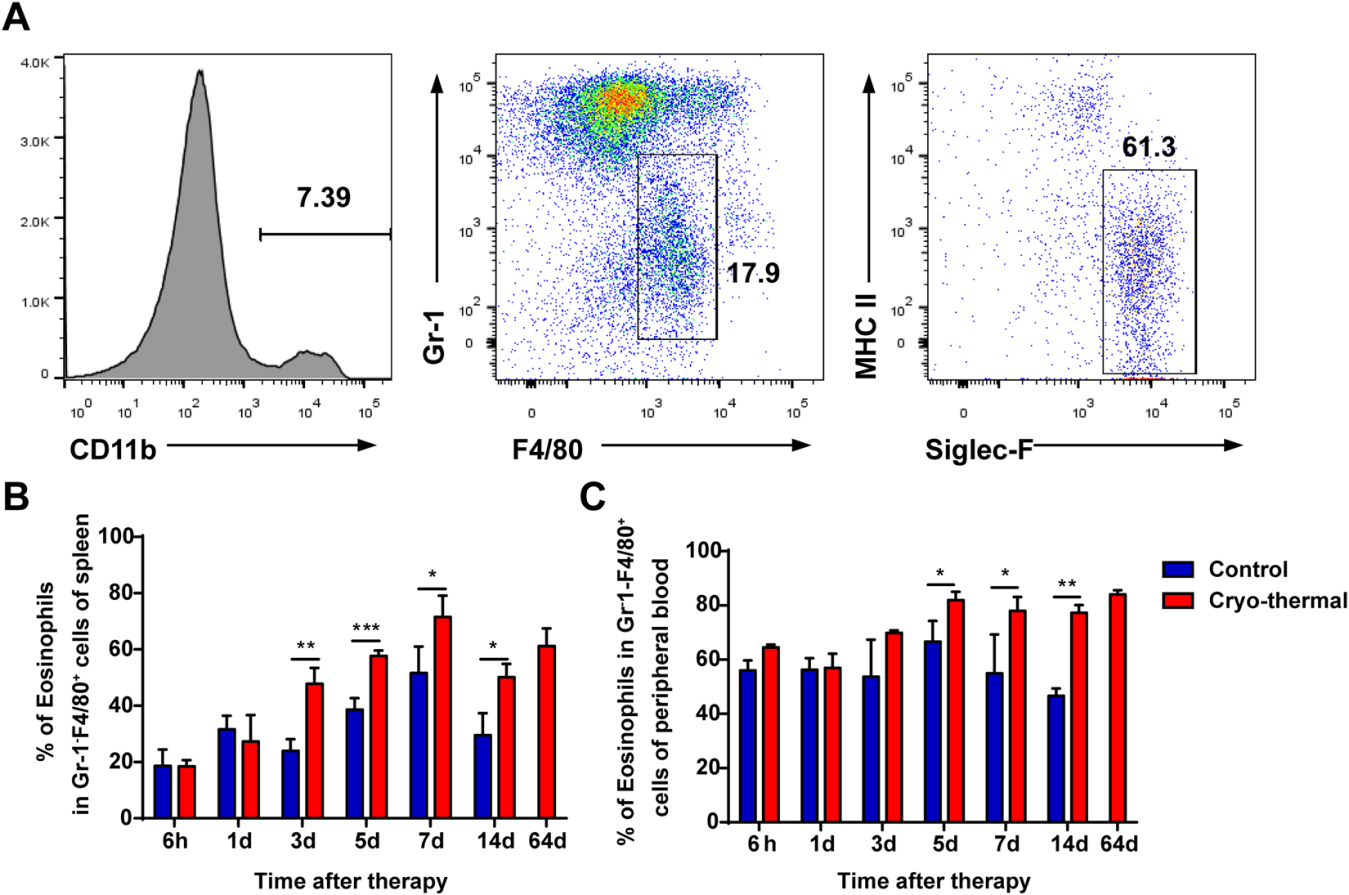
Cryo-thermal therapy induced increase of the proportion of eosinophils in spleen and peripheral blood. The phenotype of immune cells harvested from the spleen and peripheral blood in cryo-thermal-treated mice and tumour-bearing mice was analyzed by flow cytometry. (**A**) Flow cytometry gating strategy for determination of eosinophils in spleen and peripheral blood. Flow-cytometry analysis of the dynamic change of eosinophils (CD11b^+^Gr-1^−^F4/80^+^MHC II^−^Siglec-F^+^) in spleen (**B**) and peripheral blood (**C**) was performed at different time points (6 h, 1d, 3d, 5d, 7d, 14d and 64d after the cryo-thermal therapy), as compared to the tumour-bearing control group. n = 4 mice at each time point per group. Data was shown as mean ± SD. Data for bar graphs was calculated using student’s t-test. *p < 0.05; **p < 0.01; ***p < 0.001.

In our previous study, the therapeutic effect of cryo-thermal therapy was clearly demonstrated using mice bearing subcutaneous 4T1 murine mammary carcinoma and murine B16F10 melanoma with long-term survival rates of over 70% and 80%, respectively^13,29^. In this study, we also repeated to study the therapeutic effect of this therapy, and survival rates in murine B16F10 melanoma was over 80% (Supplementary Fig. S1). To comprehensively investigate the potential role of eosinophils on anti-tumour immunity elicited by local cryo-thermal therapy, a time-course study was carried out to investigate the changes of eosinophils after cryo-thermal therapy by using flow cytometry. Eosinophils were characterized as CD11b^+^Gr-1^−^F4/80^+^MHC II^−^Siglec-F^+^ cells (Fig. 1A). The percentage of eosinophils in spleen and the peripheral blood was analyzed (Fig. 1B,C). The proportion of eosinophils in spleen was obviously increased on day 3, and continuously increased in spleen and the peripheral blood on day 5, 7, 14 after cryo-thermal therapy, then eosinophils eventually kept at a relatively high level on day 64. The result showed that cryo-thermal therapy induced a marked increase of eosinophils in spleen from day 3 after the treatment.

**Figure 2:**
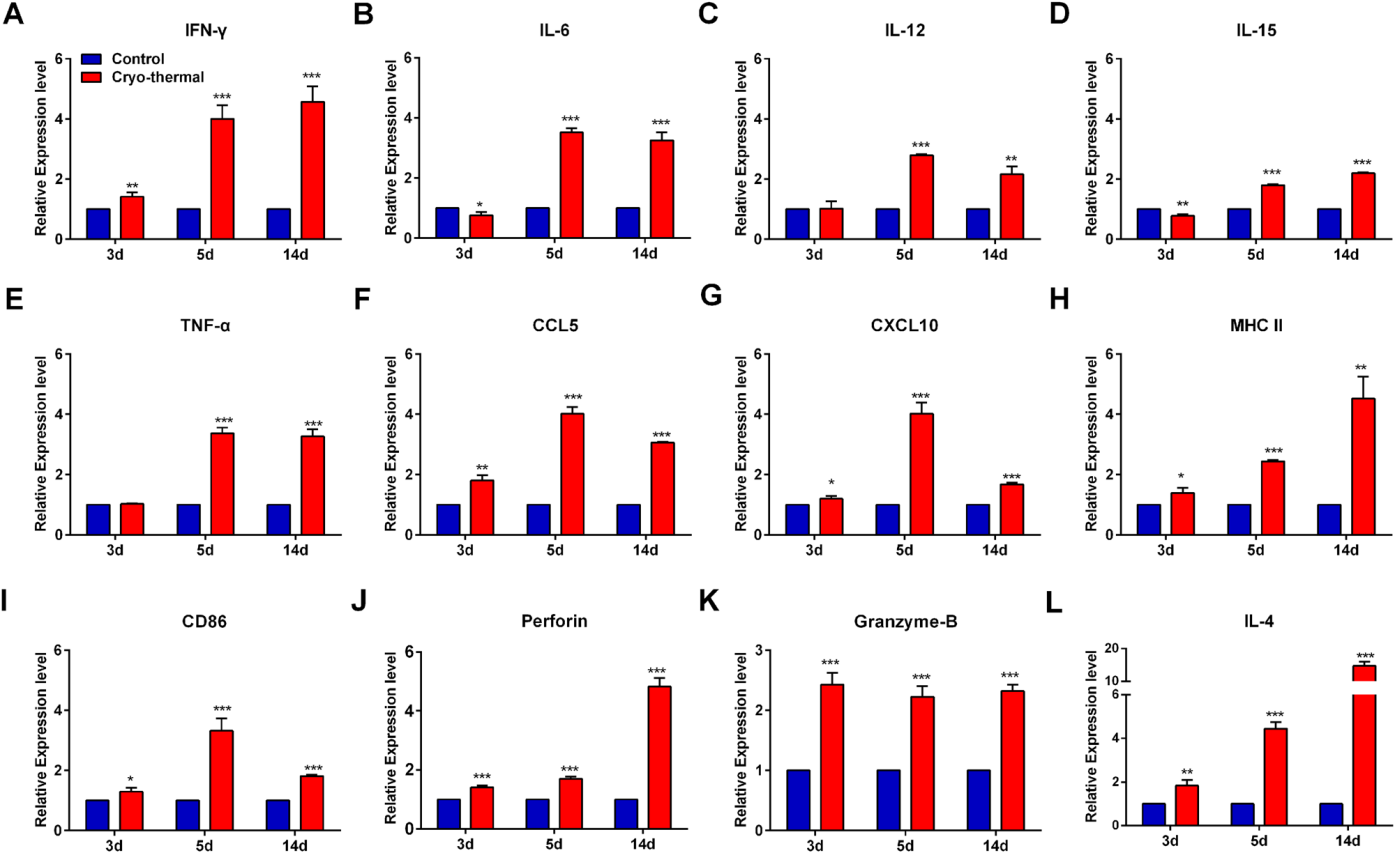
Cryo-thermal therapy induced the activation of eosinophils. Total RNA was isolated from splenic Siglec-F^+^ eosinophils in cryo-thermal-treated mice on day 3, 5 and 14 after the treatment. The mRNA in splenic Siglec-F^+^ eosinophils from tumour-bearing mice on day 15, 17 and 26 after tumour inoculation was used as control. The expression level of IFN-γ (**A**), IL-6 (**B**), IL-12 (**C**), IL-15 (**D**), TNF-α (**E**), CCL5 (**F**), CXCL10 (**G**), MHC II (**H**), CD86 (**I**), Perforin (**J**), Granzyme-B (**K**) and IL-4 (**L**). Data was shown as mean ± SD. Data for bar graphs was calculated using student’s t-test. *p < 0.05; **p < 0.01; ***p < 0.001.

To evaluate the phenotype of eosinophils induced by cryo-thermal therapy, mRNA expression of cytokines, chemokines, cytolytic molecules, and co-stimulatory molecules in sorted splenic Siglec-F^+^ eosinophils on day 3, 5 and 14 after the treatment was evaluated by RT-qPCR. On day 3, 5 and 14 after cryo-thermal therapy, the relative mRNA expression of IFN-γ was significantly up-regulated (Fig. 2A). The level of other pro-inflammatory cytokines IL-12 and TNF-α was not changed, while the mRNA expression of IL-6 and IL-15 was down-regulated on day 3, but all significantly up-regulated on day 5 and 14 (Fig. 2B–E). Meanwhile, cryo-thermal therapy induced a continuously up-regulation on the mRNA expression level of chemokines (CCL5 and CXCL10), co-stimulatory molecules (MHC II and CD86) and cytotoxic molecules (perforin and granzyme-B) was continuously increased on day 3, 5, 14 (Fig. 2F–K). Furthermore, IL-4, an essential cytokine for Th2 immune response, was also up-regulated on day 3, 5, 14 (Fig. 2L). These findings indicated that cryo-thermal therapy effectively enhanced the pro-inflammatory, antigen presenting, chemotactic and cytolytic function of eosinophils, but also induced eosinophils to highly express the anti-inflammatory cytokine.

### Cryo-thermal therapy enhanced the cytotoxic potential of eosinophils

**Figure 3:**
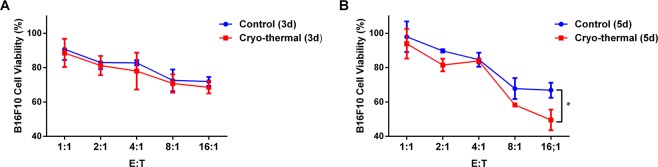
Cryo-thermal therapy enhanced the cytotoxic activity of eosinophils against tumour cells. Cytotoxic potential of eosinophils after cryo-thermal therapy. Splenic Siglec-F^+^ eosinophils in cryo-thermal-treated mice and tumour-bearing mice were purified and co-cultured with B16F10 cells at indicated ratios, and the cell viability (%) of B16F10 cells was determined by a CCK-8 assay. (**A**) The B16F10 cell viability (%) in co-cultured with eosinophils from cryo-thermal-treated mice on day 3 or tumour-bearing mice on day 15 after tumour inoculation. (**B**) The B16F10 cell viability (%) in co-cultured with eosinophils from cryo-thermal-treated mice on day 5 or tumour-bearing mice on day 17 after tumour inoculation. Data was shown as mean ± SD. Data for bar graphs was calculated using student’s t-test. *p < 0.05.

The above results revealed that eosinophils highly expressed cytotoxic proteins (perforin and granzyme-B) on day 3, 5, 14 after cryo-thermal therapy. For further verification, the cytotoxicity of splenic eosinophils against B16F10 melanoma cells was tested *in vitro*. B16F10 melanoma cells were co-cultured with eosinophils from cryo-thermal-treated or tumour-bearing mice at various ratios for 24 h, and cell viability (%) was evaluated by CCK-8. On day 3 following the treatment, there was no significant difference in cytolysis of eosinophils from cryo-thermal or tumour-bearing mice co-cultured with B16F10 cells (Fig. 3A). But on day 5, eosinophils isolated from cryo-thermal-treated mice were co-cultured with B16F10 cells at ratios of 16:1, and the cell viability of B16F10 cells was significantly decreased as compared to B16F10 cells co-cultured with tumour-bearing eosinophils (Fig. 3B). The result revealed that cryo-thermal therapy could enhance the cytotoxic potential of eosinophils.

### Activation of eosinophils after cryo-thermal therapy induced DCs maturation and macrophage polarization toward M1 phenotype

**Figure 4:**
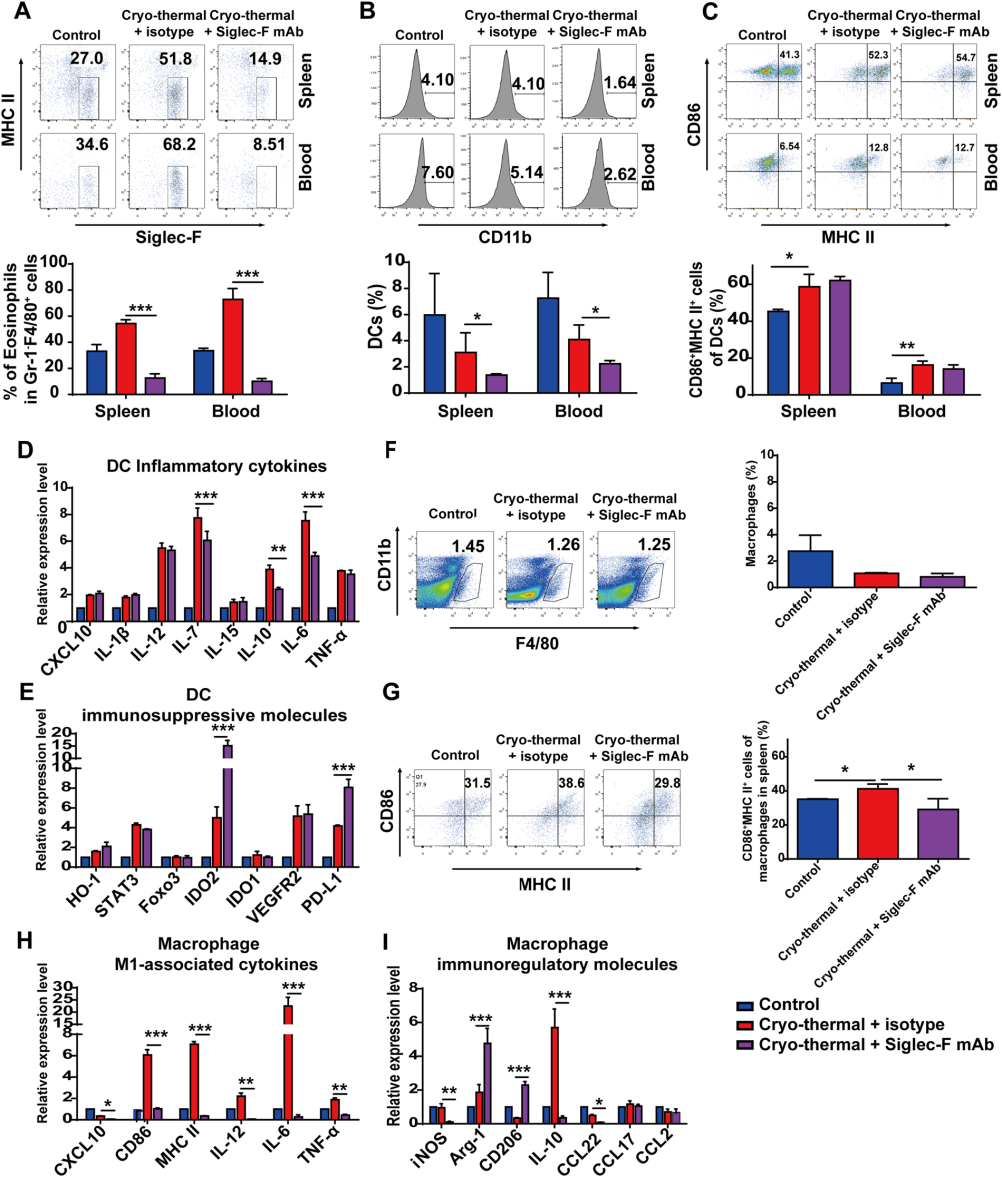
Cryo-thermal-activated eosinophils induced DCs maturation and macrophage polarization toward M1 phenotype. (**A**) The efficiency of eosinophils depletion was evaluated by flow cytometry assay. (**B**,**C**) The splenocytes and peripheral blood mononuclear cells were harvested from mice in cryo-thermal therapy + isotype, cryo-thermal therapy + Siglec-F mAb and control groups on day 14 after the treatment, then the population of total CD11c^+^ cells and the percentage of CD86^+^MHC II^+^ cells in CD11c^+^DCs were analyzed by flow cytometry. (**D**,**E**) Total RNA was isolated from splenic CD11c^+^ DCs in cryo-thermal therapy + isotype, cryo-thermal therapy + Siglec-F mAb and control groups on day 14 after the treatment. RT-qPCR analysis was performed to measure the level of pro-inflammatory cytokines and immunosuppressive molecules. (**F**,**G**) The population of total CD11b^+^F4/80^+^ cells and the percentage of CD86^+^MHC II^+^ cells in CD11b^+^F4/80^+^ macrophages were analyzed by flow cytometry. (**H,I**) Total RNA was isolated from splenic CD68^+^ macrophages in cryo-thermal therapy + isotype, cryo-thermal therapy + Siglec-F mAb and control groups on day 14 after the treatment. RT-qPCR analysis was performed to measure the level of M1-associated cytokines and immunoregulatory molecules. n = 4 mice per group. Data was shown as mean ± SD. Data for bar graphs was calculated using student’s t-test (**A**–**C**,**F**,**G**) or two-way ANOVA (**D**,**E**,**H**,**I**). *p < 0.05; **p < 0.01; ***p < 0.001.

Eosinophils can enhance DCs maturation and regulate the functional polarization of macrophage^26,27^. The above results revealed that eosinophils were activated from day 3 after cryo-thermal therapy (Fig. 1A,B), moreover, there were no significant difference in the proportion of splenic matured DCs (CD11c^+^CD86^+^MHC II^+^) and M1 macrophage (CD11b^+^F4/80^+^CD86^+^MHCII^+^) on day 3 following cryo-thermal therapy (Supplementary Fig. S2). Our previous study showed that cryo-thermal therapy markedly triggered M1 macrophage polarization and promoted DCs maturation from day 5 to 14 after the treatment (submitted). That was, the time window of eosinophils activation was earlier than macrophage polarization and DCs maturation. In this regard, we speculated that cryo-thermal therapy induced the activation of eosinophils early after the treatment, which would affect subsequently M1 macrophage polarization, DCs maturation leading to elicit systemic anti-tumour immune response. To address this hypothesis, anti-Siglec-F monoclonal antibody was used to deplete eosinophils via the induction of apoptosis, and the depletion of eosinophils was confirmed *in vivo* on day 14 after cryo-thermal therapy (Fig. 4A).

We first evaluated the changes of phenotype and function of splenic DCs after eosinophils depletion by flow cytometry and RT-qPCR, respectively. A significant decrease of the total CD11c^+^ population in spleen and blood was found on day 14 following cryo-thermal therapy with Siglec-F mAb treatment as compared to cryo-thermal therapy + isotype group (Fig. 4B). The matured DCs (CD11c^+^CD86^+^MHC II ^+^DCs) in spleen and blood were up-regulated after cryo-thermal therapy (Fig. 4C). However, there was no difference between cryo-thermal therapy + Siglec-F mAb and cryo-thermal therapy + isotype groups. Meanwhile, the mRNA expression of pro-inflammatory cytokines IL-7, IL-10 and IL-6 in splenic DCs after cryo-thermal therapy with Siglec-F mAb treatment was markedly reduced as compared to cryo-thermal therapy + isotype group (Fig. 4D). Unexpectedly, there was no difference in the expression of other pro-inflammatory cytokines, such as CXCL10, IL-12, IL-1β, IL-15 and TNF-α between two groups. Moreover, mRNA expression level of immunosuppressive molecules, IDO2 and PD-L1 were significantly up-regulated after cryo-thermal therapy with Siglec-F mAb treatment as compared to cryo-thermal therapy + isotype group, and the other immunosuppressive molecules HO-1, STAT3, Foxo3, IDO1 and VEGFR2 was not obviously changed in two groups (Fig. 4E). The result indicated that cryo-thermal-activated eosinophils could partly induce the activation and maturation of DCs.

Then, the phenotype and function of macrophage were also analyzed after eosinophils depletion by flow cytometry and RT-qPCR, respectively. Flow cytometry analysis showed that eosinophils depletion did not affect the proportion of macrophage (CD11b^+^F4/80^+^) in spleen and blood, but resulted in less M1 macrophage population (CD11b^+^F4/80^+^CD86^+^MHCII^+^) as compared to cryo-thermal therapy + isotype group (Fig. 4F,G). More importantly, the gene expression of M1-associated markers, CXCL10, CD86, MHCII, IL-12, IL-6 and TNF-α was significantly down-regulated in splenic macrophage after cryo-thermal therapy + Siglec-F mAb treatment as compared to cryo-thermal therapy + isotype group (Fig. 4H). A markedly up-regulated expression of the immunoregulatory molecules, Arg-1 and CD206 after cryo-thermal therapy + Siglec-F mAb treatment was found. Meanwhile, the expression level of IL-10, iNOS and CCL22 in cryo-thermal therapy + Siglec-F mAb group was significantly decreased as compared to that in cryo-thermal therapy + isotype group, and the level of CCL17 and CCL2 were unchanged in two groups (Fig. 4I). The result showed that cryo-thermal-activated eosinophils could effectively induce M1 macrophage polarization, moreover, the effect of cryo-thermal-activated eosinophils on M1 macrophage polarization was much stronger than the effect of eosinophils on DCs activation and maturation.

### Cryo-thermal-activated eosinophils modulated the phenotype and function of macrophages and DCs by IL-4 and IFN-γ *in vitro*

The above results demonstrated that eosinophils highly expressed various cytokines, chemokines and co-stimulatory molecules from day 3 to 14 after cryo-thermal therapy (Fig. 2). Among them, CCL5 and CXCL10 are known to be potent chemo-attractants for other immune cells, especially T cells^30^. Granzyme-B and Perforin represented the cytotoxic capacity of eosinophils. The increased expression of co-stimulatory molecules CD86 and MHC II in eosinophils indicated that cryo-thermal therapy effectively enhanced antigen-presentation function of eosinophils, while the antigen-presentation function is also modulated by various pro-inflammatory signals, such as IL-6, IL-12, IL-15 and TNF-α^25^. However, the role of high expression of IFN-γ and IL-4 in cryo-thermal-activated eosinophils remained unresolved. Th1 cytokine IFN-γ plays a pivotal role in anti-tumor immune response, which can regulate the function of other immune cells, including DCs, macrophage and T cells^31–33^. IL-4 is a typical Th2 cytokine. Eosinophils could modulate macrophage phenotype in an IL-4 dependent manner^27^. Considering the above results that cryo-thermal-activated eosinophils could induce M1 macrophage polarization and DCs maturation, we hypothesized whether the high expression of IFN-γ and IL-4 in cryo-thermal-activated eosinophils played a vital role in modulating the phenotype and function of DCs and macrophage. To address the hypothesis, we confirmed the high-level expression of IFN-γ and IL-4 in eosinophils on day 3 and 14 after cryo-thermal therapy by flow cytometry (Supplementary Fig. S3).

**Figure 5:**
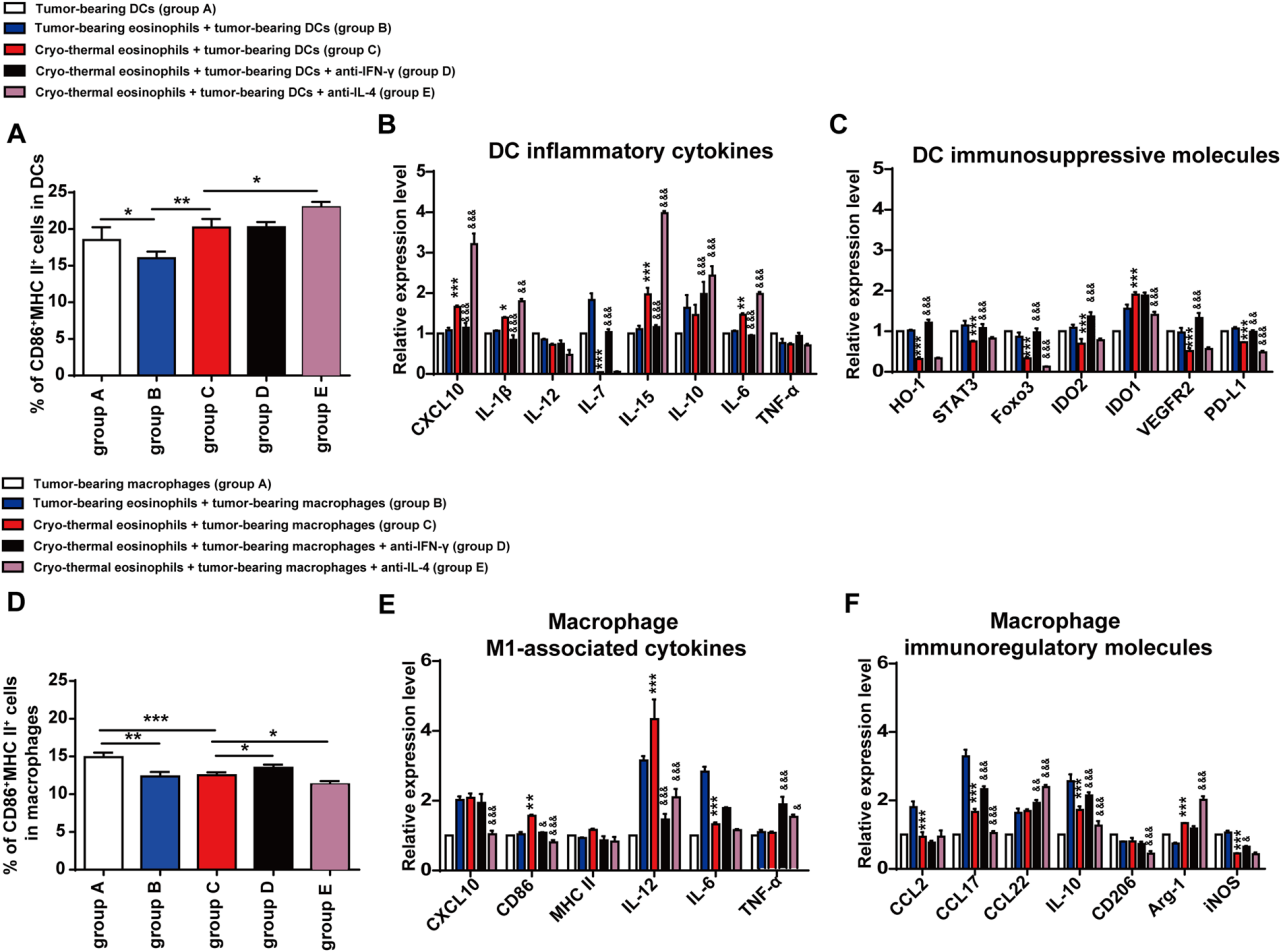
Cryo-thermal-activated eosinophils modulated the phenotype and function of macrophages and DCs by IL-4 and IFN-γ *in vitro*. (**A**) The percentage of CD86^+^MHC II^+^ cells in CD11c^+^DCs after co-cultured with eosinophils were analyzed by flow cytometry. The tumour-bearing CD11c^+^ DCs were used as control. (**B**,**C**) Total RNA was isolated from CD11c^+^ DCs after co-cultured with eosinophils and RT-qPCR analysis was performed to measure the level of pro-inflammatory cytokines and immunosuppressive molecules. (**D**) The percentage of CD86^+^MHC II^+^ cells in F4/80^+^ macrophages after co-cultured with eosinophils were analyzed by flow cytometry. The tumour-bearing macrophages were used as control. (**E**,**F**) Total RNA was isolated from macrophages after co-cultured with eosinophils and RT-qPCR analysis was performed to measure the level of M1-associated cytokines and immunoregulatory molecules. Data was shown as mean ± SD. Data for bar graphs was calculated using student’s t-test (**A**,**D**) or two-way ANOVA (**B**,**C**,**E**,**F**). *p < 0.05; **p < 0.01; ***p < 0.001 was considered to be statistically significant compared to that in tumour-bearing eosinophils + tumour-bearing DCs/macrophage group (**B**,**C**,**E**,**F**). ^&^p < 0.05; ^&&^p < 0.01; ***p < 0.001 was considered to be statistically significant compared to that in cryo-thermal eosinophils + tumour-bearing DCs/macrophage group (**B**,**C**,**E**,**F**).

We next verified the effect of IFN-γ and IL-4 derived from cryo-thermal-activated eosinophils on modulating DCs maturation *in vitro*. The splenic CD11c^+^ DCs were isolated from tumour-bearing mice on day 15 following tumour inoculation (termed as tumour-bearing DCs). The splenic eosinophils were isolated from tumour-bearing mice on day 15 following tumour inoculation (termed as tumour-bearing eosinophils), and cryo-thermal treated mice on day 3 (termed as cryo-thermal eosinophils). The tumour-bearing DCs alone was as the control. The tumour-bearing eosinophils were co-cultured with tumour-bearing DCs at a ratio of 1:1 for 24 h (tumour-bearing eosinophils + tumour-bearing DCs). Meanwhile, cryo-thermal eosinophils were co-cultured with tumour-bearing DCs, adding isotype IgG1 (cryo-thermal eosinophils + tumour-bearing DCs), IFN-γ neutralizing antibody (cryo-thermal eosinophils + tumour-bearing DCs + anti IFN-γ, termed as IFN-γ neutralizing group), IL-4 neutralizing antibody (cryo-thermal eosinophils + tumour-bearing DCs + anti-IL-4 antibody, termed as IL-4 neutralizing group), respectively. We analyzed the phenotype and function of DCs by using flow cytometry and RT-qPCR, respectively. The proportion of total CD11c^+^ cells was not significant difference in all the groups (Supplementary Fig. S4A). However, the percentage of matured DCs (CD11c^+^CD86^+^ MHC II ^+^) was significantly down-regulated when DCs were co-cultured with tumour-bearing eosinophils as compared to DCs cultured alone. Interestingly, higher level of matured DCs was induced when tumour-bearing DCs were co-cultured with cryo-thermal eosinophils as compared to tumour-bearing DCs co-cultured with tumour-bearing eosinophils. The percentage of matured DCs in IL-4 neutralizing group was significantly increased as compared to cryo-thermal eosinophils + tumour-bearing DCs group, but blockade of IFN-γ had no difference (Fig. 5A). Furthermore, when tumour-bearing DCs were co-cultured with cryo-thermal eosinophils, the expression level of pro-inflammatory cytokines (CXCL10, IL-1β, IL-15 and IL-6) was increased as compared to tumour-bearing DCs co-cultured with tumour-bearing eosinophils. The other (IL-12, IL-10 and TNF-α) was not affected and the expression of IL-7 was down-regulated (Fig. 5B). Moreover, tumour-bearing DCs co-cultured with cryo-thermal eosinophils resulted in a significant down-regulation of immunosuppressive molecules, HO-1, STAT3, Foxo3, IDO2, VEGFR2 and PD-L1 as compared to tumour-bearing DCs co-cultured with tumour-bearing eosinophils. Unexpectedly, the expression level of IDO1 was increased (Fig. 5C). Meanwhile, after the addition of IFN-γ neutralizing antibody, the relative expression level of pro-inflammatory cytokines (CXCL10, IL-1β, IL-15 and IL-6) was significantly reduced, and the gene expression of immunosuppressive molecules (HO-1, STAT3, Foxo3, IDO2, VEGFR2 and PD-L1) was increased as compared to cryo-thermal eosinophils + tumour-bearing DCs group, in addition, the level of IL-7 and IL-10 were up-regulated (Fig. 5B,C). The results indicated that the high expression of IFN-γ in cryo-thermal-activated eosinophils could promote DCs activation and maturation. Moreover, blockade of IL-4 resulted in overexpression of pro-inflammatory cytokines (CXCL10, IL-1β, IL-15, IL-10 and IL-6) and lower expression of immunosuppressive molecules (Foxo3, IDO1 and PD-L1) as compared to cryo-thermal eosinophils + tumour-bearing DCs group (Fig. 5B,C). The results showed that the high expression of IL-4 in cryo-thermal-activated eosinophils could inhibit overexpression of pro-inflammatory cytokines and promote expression of immunosuppressive molecules in DCs. All these results revealed that cryo-thermal-activated eosinophils could promote the activation and maturation of DCs through IFN-γ and IL-4 exerting the antagonistic effects.

Then we investigated the role of IFN-γ and IL-4 in cryo-thermal-activated eosinophils on macrophage polarization *in vitro*. The splenic CD68^+^ macrophages were isolated from tumour-bearing mice on day 15 following tumour inoculation (termed as tumour-bearing macrophage). Siglec-F^+^ eosinophils were isolated from tumour-bearing mice on day 15 following tumour inoculation (termed as tumour-bearing eosinophils), and cryo-thermal treated mice on day 3 (termed as cryo-thermal eosinophils). The groups of *in vitro* assays were listed as follows: tumour-bearing macrophage alone was considered as the control group; tumour-bearing eosinophils + tumour-bearing macrophage group; cryo-thermal eosinophils + tumour-bearing macrophage group; cryo-thermal eosinophils + tumour-bearing macrophage + anti-IFN-γ antibody, termed as IFN-γ neutralizing group; cryo-thermal eosinophils + tumour-bearing macrophage + anti-IL-4 antibody, termed as IL-4 neutralizing group. The phenotype and function of co-cultured macrophages were analyzed by using flow cytometry and RT-qPCR, respectively. The percentage of total F4/80^+^ macrophages was higher in IL-4 neutralizing group as compared to cryo-thermal eosinophils + tumour-bearing macrophage group (Supplementary Fig. S4B). Meanwhile, macrophages co-cultured with eosinophils from tumour-bearing mice or cryo-thermal treated mice both resulted in a decrease of M1 macrophage (CD11b^+^F4/80^+^CD86^+^MHCII^+^) population as compared to tumour-bearing macrophages alone. Blockade of IFN-γ with neutralizing antibodies resulted in slightly high proportion of M1 macrophage compared with that in cryo-thermal eosinophils + tumour-bearing macrophage group, and IL-4 neutralizing reduced it (Fig. 5D). Furthermore, when macrophages were co-cultured with cryo-thermal eosinophils, the expression level of M1-associated co-stimulatory molecules and cytokines, CD86 and IL-12 were significantly up-regulated as compared to macrophages co-cultured with tumour-bearing eosinophils, and the expression of IL-6 was down-regulated (Fig. 5E). More importantly, immunoregulatory molecules (CCL2, CCL17, IL-10 and iNOS) were obviously down-regulated when macrophages were co-cultured with cryo-thermal eosinophils. However, Arg-1 was up-regulated (Fig. 5F). After IFN-γ neutralizing, the gene expression of M1-associated co-stimulatory molecules and cytokines (CD86 and IL-12) was reduced and the expression of immunoregulatory molecules (CCL17, CCL22, IL-10 and iNOS) was higher than cryo-thermal eosinophils + tumour-bearing macrophage group, though the expression of M1-associated cytokines TNF-α was increased (Fig. 5E,F). After blockade of IL-4, the down-regulation of M1-associated cytokines (CXCL10, CD86, and IL-12) was observed as compared to cryo-thermal eosinophils + tumour-bearing macrophage group. However, the expression level of TNF-α was higher. Meanwhile, IL-4 neutralization resulted in a decrease of immunoregulatory molecules (CCL17, IL-10 and CD206), but CCL22 and Arg-1 were up-regulated (Fig. 5E,F). These results revealed that cryo-thermal-activated eosinophils not only promoted macrophage polarization toward M1 phenotype by additive or synergistic effect of IFN-γ and IL-4, but also modulated the expression of some immunosuppressive molecules (CCL17, IL-10) in macrophages by the antagonistic effect of IFN-γ and IL-4. Moreover, IL-4 derived from cryo-thermal-activated eosinophils could inhibit overexpression of CCL22 and Arg-1 in macrophages after cryo-thermal therapy.

### Cryo-thermal-activated eosinophils induced the functional differentiation of CD4^+^ T cells and development of cytotoxic CD8^+^ T cells

**Figure 6:**
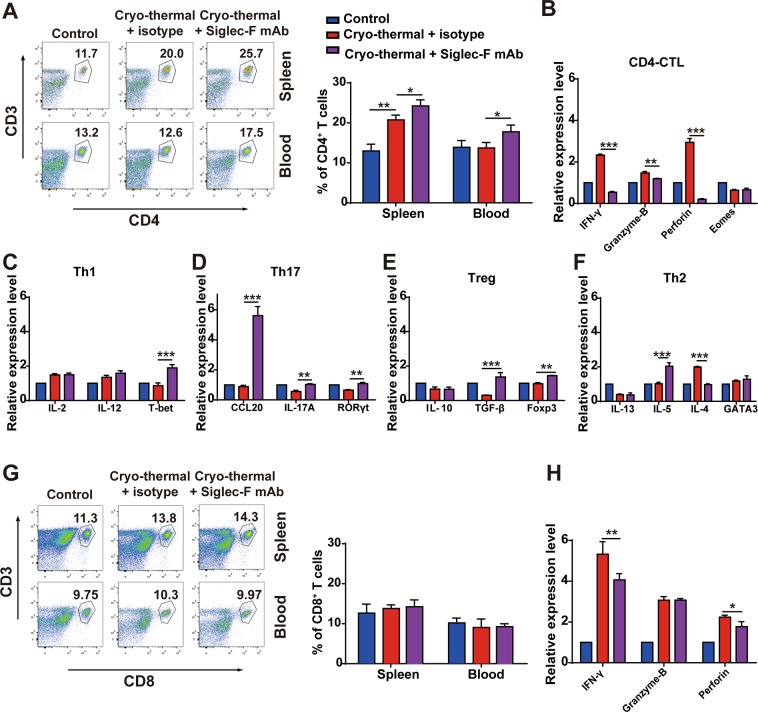
Cryo-thermal-activated eosinophils promoted the differentiation of CD4^+^ T cells and generation of cytotoxic CD8^+^ T cells. (**A**) The splenocytes and peripheral blood mononuclear cells were harvested from mice in cryo-thermal therapy + isotype, cryo-thermal therapy + Siglec-F mAb and control groups on day 14 after the treatment, and the percentage of CD3^+^CD4^+^ T cells were analyzed by flow cytometry. The expression of CD4-CTL (**B**), Th1 (**C**), Th17 (**D**), Treg (**E**) and Th2 (**F**) cell subsets in splenic CD4^+^ T cells were assayed by RT-qPCR. (**G**) The splenocytes and peripheral blood mononuclear cells were harvested from mice in cryo-thermal therapy + isotype, cryo-thermal therapy + Siglec-F mAb and control groups on day 14 after the treatment, and the percentage of CD3^+^CD8^+^ T cells were analyzed by flow cytometry. (**H**) The expression of IFN-γ, perforin and granzyme-B in splenic CD8^+^ T cells were assayed by RT-qPCR. n = 4 mice per group. Data was shown as mean ± SD. Data for bar graphs was calculated using student’s t-test (**A**,**G**) or two-way ANOVA (**B**–**F**,**H**). *p < 0.05; **p < 0.01; ***p < 0.001.

Previous studies indicate that activated eosinophils can present antigens to CD4^+^ T cells and promote the functional polarization of CD4^+^ T cells^34^. Meanwhile, the activated eosinophils can promote the infiltration of CD8^+^ T cells into tumour^28^. To research the role of cryo-thermal-activated eosinophils in long-lasting anti-tumour immunity mediated by cryo-thermal therapy, the subsets of T cells were evaluated after cryo-thermal therapy with eosinophils depletion. The level of splenic total CD4^+^ T cells and sub-lineages of CD4^+^ T-cells were analyzed by using flow cytometry and RT-qPCR, respectively. The percentages of CD4^+^ T cells in spleen and blood was significantly increased after cryo-thermal therapy with eosinophils depletion treatment as compared to cryo-thermal therapy + isotype group (Fig. 6A). Meanwhile, the gene expression of CD4-CTL associated cytokines (Perforin, Granzyme-B, IFN-γ) in splenic CD4^+^ T cells were significantly decreased, though the CD4-CTL transcription factor Eomes was not affected (Fig. 6B). Th1 transcription factor T-bet was increased after cryo-thermal therapy with Siglec-F mAb treatment, but Th1-associated cytokines (IL-2, IL-12) were not changed (Fig. 6C). Interestingly, the expression level of Th17-associated cytokines (CCL20, IL-17A) and transcription factor RORγt were significantly up-regulated after cryo-thermal therapy with Siglec-F mAb treatment as compared to cryo-thermal therapy + isotype group (Fig. 6D). Meanwhile, the mRNA expression level of Treg-associated cytokine TGF-β and transcription factor Foxp3 were much higher than that in cryo-thermal therapy + isotype group (Fig. 6E). The increased expression of Th2-associated cytokine IL-5 was observed after cryo-thermal therapy with Siglec-F mAb treatment, however, the expression level of Th2-associated cytokine IL-4 was decreased (Fig. 6F). The results suggested that cryo-thermal-activated eosinophils induced the differentiation of CD4^+^ T cells into CD4-CTL, but inhibited the differentiation of Tregs and Th17 subsets after cryo-thermal therapy.

Moreover, we also evaluated the percentage and cytotoxicity of splenic CD8^+^ T cells. The proportion of CD8^+^ T cells in blood and spleen was not affected after cryo-thermal therapy with Siglec-F mAb treatment as compared to cryo-thermal therapy + isotype group (Fig. 6G). However, the mRNA level of IFN-γ and perforin were significantly down-regulated after cryo-thermal therapy with Siglec-F mAb treatment (Fig. 6H), which revealed that cryo-thermal-activated eosinophils enhanced the cytotoxic function of CD8^+^ T cells. The above results revealed that cryo-thermal-activated eosinophils could promote CD4^+^ T cells differentiate to CD4^+^ CTL cells and enhance the cytotoxicity of CD8^+^ T cells.

Then, we next evaluated the cytotoxicity of splenic CD4^+^ and CD8^+^ T cells against B16F10 melanoma cells *in vitro* after cryo-thermal therapy with Siglec-F mAb by CCK-8. B16F10 melanoma cells were cultured in the presence or absence of splenic CD4^+^ or CD8^+^ T cells from tumour-bearing mice, cryo-thermal with isotype treated mice and cryo-thermal with Siglec-F mAb treated mice at various ratios for 24 h, and the cell viability (%) was determined. There was no significant difference in cytolysis of splenic CD8^+^ T cells co-cultured with B16F10 cells at effect-to-target cell ratio of 1:2 in three groups. However, when CD8^+^ T cells from cryo-thermal + Siglec-F mAb treated mice were co-cultured with B16F10 cells at ratio of 1:1, the cell viability of B16F10 cells was significantly increased as compared to cryo-thermal + isotype groups. More interestingly, when CD8^+^ T cells from cryo-thermal + Siglec-F mAb treated mice were co-cultured with B16F10 cells at ratios of 4:1 and 8:1, CD8^+^ T cells promoted the proliferation of B16F10 cells (Supplementary Fig. S5A). Meanwhile, when CD4^+^ T cells from cryo-thermal + Siglec-F mAb treated mice were cultured with B16F10 cells at various ratios, the cell viability of B16F10 cells was significantly increased as compared to cryo-thermal + isotype groups (Supplementary Fig. S5B). These results indicated that cryo-thermal-activated eosinophils could enhance the cytotoxicity of CD4^+^ and CD8^+^ T cells.

### Cryo-thermal-activated eosinophils promoted the cytolytic capacity of CD4^+^ and CD8^+^ T cells *in vitro*

**Figure 7:**
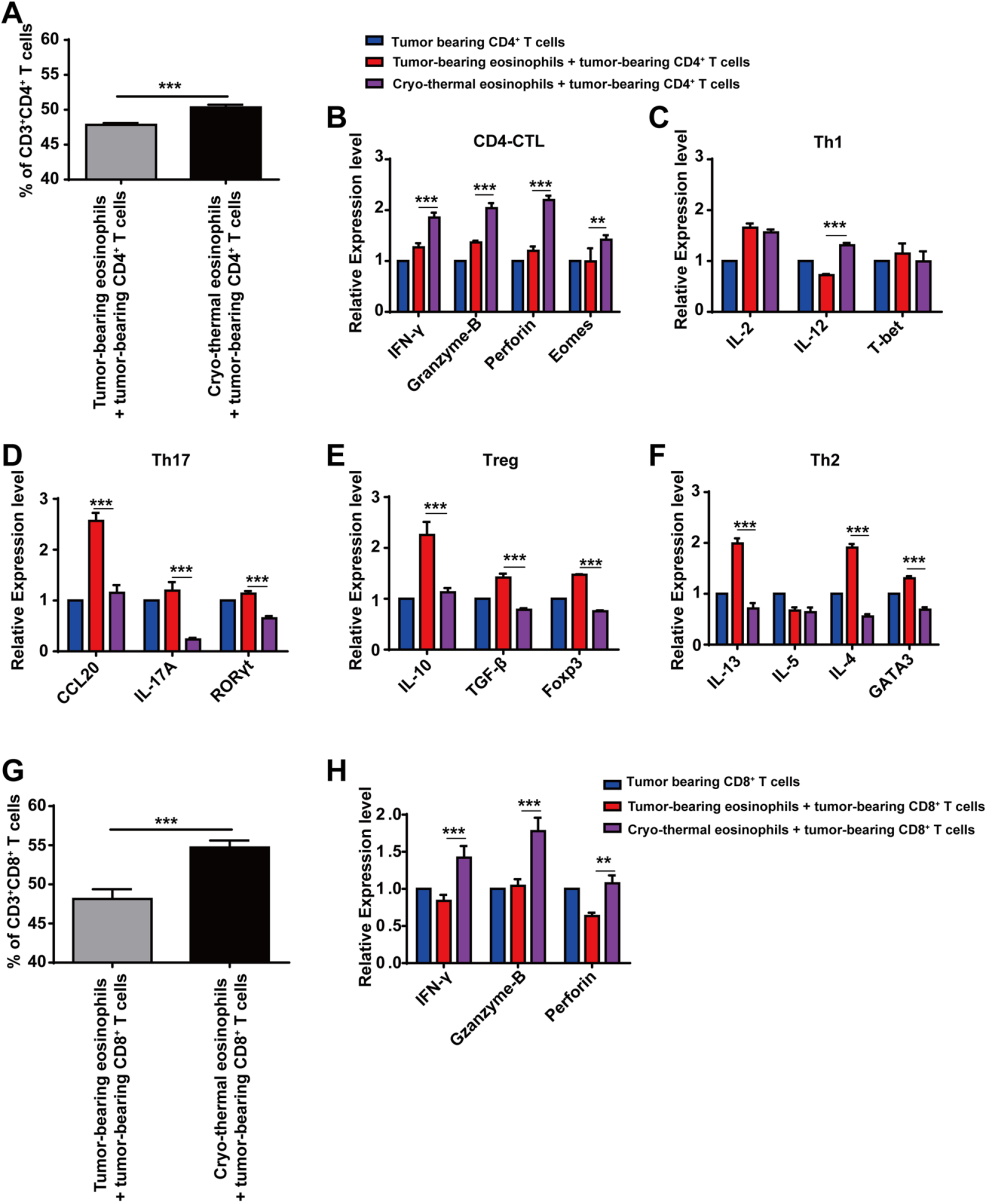
Cryo-thermal-activated eosinophils promoted the cytolytic capacity of CD4^+^ and CD8^+^ T cells *in vitro*. (**A**) The percentage of CD3^+^CD4^+^ T cells after co-cultured with eosinophils were analyzed by flow cytometry. Total RNA was isolated from CD4^+^ T cells after co-cultured with eosinophils and RT-qPCR analysis was performed to measure the level of CD4-CTL (**B**), Th1 (**C**), Th17 (**D**), Treg (**E**) and Th2 (**F**) cell subsets. (**G**) The percentage of CD3^+^CD8^+^ T cells after co-cultured with eosinophils were analyzed by flow cytometry. (**H**) Total RNA was isolated from CD8^+^ T cells after co-cultured with eosinophils and RT-qPCR analysis was performed to measure the level of IFN-γ, perforin and granzyme-B. Data was shown as mean ± SD. Data for bar graphs was calculated using student’s t-test (**A**,**G**) or two-way ANOVA (B-F, H). *p < 0.05; **p < 0.01; ***p < 0.001.

Considering the above results that cryo-thermal-activated eosinophils was the key mediator of CD4^+^ T cells differentiation and cytotoxic CD8^+^ T cells development, whether the polarization of CD4^+^ T cells and cytotoxic CD8^+^ T cells was regulated through direct interaction with cryo-thermal-activated-eosinophils would be addressed. Therefore, we performed *in vitro* experiments to research the direct impact of cryo-thermal-activated eosinophils on the phenotype and function of CD4^+^ and CD8^+^ T cells. Splenic CD4^+^ T cells were isolated from tumour-bearing mice on day 15 following tumour inoculation, and co-cultured with eosinophils from tumour-bearing mice on day 15 following tumour inoculation or cryo-thermal treated mice on day 3 at a ratio of 5:1 for 24 h. The tumour-bearing CD4^+^ T cells co-cultured with tumor-bearing eosinophils was as the control. The proportion of co-cultured CD4^+^ T cells and markers of CD4^+^ T-cell subsets were tested by flow cytometry and RT-qPCR, respectively. As compared to the control group, co-cultured with cryo-thermal-activated eosinophils significantly increased the level of total CD4^+^ T cells (Fig. 7A). Furthermore, the gene expression of CD4-CTL associated cytokines (IFN-γ, granzyme-B, perforin) and transcription factor Eomes were significantly higher (Fig. 7B). The expression of Th1-associated cytokines IL-12 was also increased, though IL-2 and T-bet were not affected (Fig. 7C). More importantly, the Th2, Th17 and Tregs related cytokines and transcription factors were all down-regulated after CD4^+^ T cells were co-cultured with cryo-thermal-activated eosinophils, though the expression of IL-5 was not changed (Fig. 7D–F). The result revealed that cryo-thermal-activated eosinophils could directly induce CD4^+^ T cells differentiate into CD4-CTL, but inhibit the differentiation of Th2, Th17 and Tregs subset, which promote the cytolytic capacity of CD4^+^ T cells.

Then the direct impact of cryo-thermal-activated eosinophils on the cytotoxicity of CD8^+^ T cells was evaluated. The CD8^+^ T cells in spleen was isolated from tumour-bearing mice on day 15 following tumour inoculation. The isolated splenic eosinophils from tumour-bearing mice on day 15 following tumour inoculation, or cryo-thermal treated mice on day 3 was co-cultured with tumour-bearing CD8^+^ T cells at a ratio of 1:5 for 24 h, respectively. Then the proportion and cytotoxic effector function of splenic CD8^+^ T cells was detected using flow cytometry and RT-qPCR. After tumour-bearing CD8^+^ T cells were co-cultured with cryo-thermal-activated eosinophils, the percentage of CD8^+^ T cells was significantly increased (Fig. 7G). Meanwhile, the mRNA level of perforin, granzyme-B and IFN-γ was significantly up-regulated (Fig. 7H). These results suggest that cryo-thermal-activated eosinophils could strongly enhance the cytolytic capacity of CD8^+^ T cells. All the data indicated that cryo-thermal-activated eosinophils could directly enhanced CD4^+^ T cells differentiate into CTL subset and the cytolytic capacity of CD8^+^ T cells.

**Figure 8:**
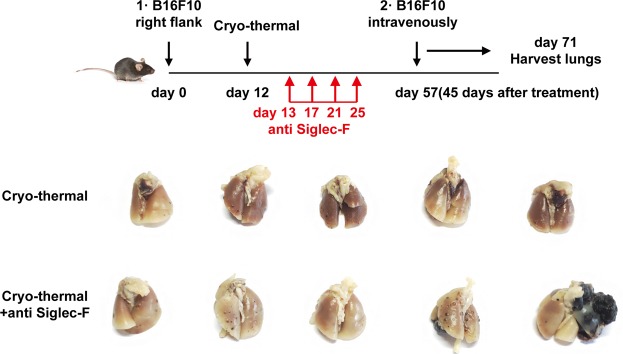
Cryo-thermal-activated eosinophils protected mice from pulmonary metastatic B16F10 tumors. Photographic images of lungs from cryo-thermal treated and cryo-thermal therapy with eosinophils depleted B16F10 tumor-bearing mice, respectively. Upper: schematic of experimental design. Lower: photographic images of lungs.

To further verify the effect of eosinophils on cryo-thermal-induced anti-tumour immunity, eosinophils were depleted after cryo-thermal therapy. 45 days after the therapy, both eosinophils depletion after cryo-thermal therapy and single cryo-thermal therapy resulted in the death of one mouse due to recurrence and lung metastasis. And the living mice in both groups were rechallenged with B16F10 melanoma cells. B16F10 melanoma cells were intravenously infused in the living mice and lung nodules were enumerated two weeks later (Fig. 8). Lung tumor nodules were almost entire controlled in long-term survivors treated by cryo-thermal therapy, but the mice in eosinophils depletion after cryo-thermal therapy obviously developed tumor nodules in the lungs. The results indicated that eosinophils has an essential role in cryo-thermal induction of anti-tumor immune memory.

## Discussion

In our previous study, we established a novel therapeutic approach to tumour of cryo-thermal therapy^12,13,35^. Cryo-thermal therapy lead to a significant inhibition of distant lung metastasis and prolonged survival in B16F10 melanoma mouse model and 4T1 murine mammary carcinoma^12,13^. Furthermore, cryo-thermal therapy effectively reshaped the immunosuppressive to an immunostimulatory state^12^, along with M1 macrophage polarization and DCs maturation, which led to durable anti-tumour memory immunity^20^. In our study, we determined the role of eosinophils in innate and adaptive immunity modulated by cryo-thermal therapy. This study demonstrated that cryo-thermal therapy induced eosinophils activation at early stage following the treatment, along with increasing eosinophils in spleen and peripheral blood with high expression level of inflammatory cytokines, chemokines and co-stimulatory molecules, and enhanced eosinophil cytotoxicity against tumour cells. Moreover, our study revealed that cryo-thermal-activated eosinophils was required to drive M1 macrophage polarization, DCs maturation, functional differentiation of CD4^+^ T cells, and generation of cytotoxic of CD8^+^ T cells. This study also presented that versatile immunologic regulation of cryo-thermal-activated eosinophils extended from innate immunity to anti-tumour adaptive immunity.

It is generally accepted that an effective innate immunity is a prerequisite for inspiring a persistent adaptive immunity^36^. Our previous study identified that the important role of cryo-thermal-induced M1 macrophage polarization and DCs maturation in the bridge of innate and adaptive immune response (submitted). Eosinophils are typical innate immune cells which can release several cytokines and interact with various innate and adaptive immune cells upon activation^37^. Moreover, eosinophils are associated with a favorable prognosis, particularly in solid tumours^25,38^. However, the role of eosinophils in the process of cryo-thermal-induced anti-tumour immunity is unclear. In our study, cryo-thermal therapy activated eosinophils in the early stage led to the induction of strong innate and adaptive anti-tumour immune response. Considering that cryo-thermal therapy triggered strong anti-tumour memory immunity to improve the long-term survival in murine B16F10 melanoma^13^, we suggested that the activated eosinophils in early stage following cryo-thermal therapy was essential to mediate effective and durable anti-tumour immunity.

In our study, cryo-thermal therapy induced the accumulation of eosinophils into spleen and elicited fully activation of eosinophils from day 3 after the treatment. The splenic eosinophils in cryo-thermal-treated mice expressed high level of chemokines and pro-inflammatory cytokines (CCL5, CXCL10, IFN-γ, IL-6, IL-12 and IL-15), which could create immunostimulatory microenvironment contributing to anti-tumour immunity^25^. Moreover, cryo-thermal therapy improved the cytolytic activity of eosinophils, as demonstrated by the up-regulation of cytotoxic molecules (e.g. granzyme-B and perforin), and enhanced tumour*-*killing capacity on tumour cells. More importantly, cryo-thermal-activated eosinophils enhanced M1 macrophage polarization, DCs maturation, differentiation of CD4^+^ T cells into CD4-CTL subset, and generation of cytotoxic of CD8^+^ T cells. We believed that eosinophils were very important in the process of cryo-thermal-induced anti-tumour immunity from innate immunity to adaptive immunity.

DCs present antigens to T cells and provide a series of crucial signals (co-stimulatory molecules and cytokines) for T cell activation and differentiation, thereby modulate the adaptive immune response^16^. But only mature DCs can stimulate T cells and activate a strong adaptive immune response^39^. Therefore, DCs maturation was required for subsequently T cells activation and shaping of effective anti-tumour immune response. DCs maturation relies on the local microenvironment and was regulated by other immune cells^40^. CpG activated eosinophils induce DCs maturation during chronic inflammatory disease^26^. In this study, cryo-thermal-activated eosinophils played a regulatory role in induction of maturation DCs. Eosinophils depletion down-regulated the expression of pro-inflammatory cytokines (IL-7 and IL-6) and up-regulation the production of immunosuppressive molecules (IDO2 and PD-L1) in DCs, while the inflammatory cytokines were required for T cell activation and immunosuppressive molecules could induce T*-*cell tolerance^41,42^. Hence, our findings indicated that cryo-thermal-activated eosinophils could induce the activation and maturation of DCs.

Among these effector cells of innate immunity, macrophages represent the most important cell of innate immunity. First, macrophages could kill neoplastic cells by phagocytosis. Second, antigen processing by macrophages can convert the antigen into several antigenic epitopes, which can be recognized by T lymphocytes. Moreover, macrophages also secrete cytokines (TNF-α, IL‐1, IL‐6 and IL‐8) that play a variety of roles in promotion of inflammation, enhancement of phagocytosis, activation of resting T lymphocytes^43^. But M2 macrophages promote tumour invasion and metastasis^18^. Only M1 macrophages serve as a pivotal role in the activation of T cells and formation of durable anti-tumour immunity^18^. Macrophages can change their functional phenotype in response to specific environmental stimuli^44^. Hence, reversing macrophages from immunosuppressive M2 phenotype to immunostimulatory M1 phenotype is useful in promoting T cells activation and differentiation. In this study, cryo-thermal-activated eosinophils modulated M1 macrophages polarization, along with the up-regulated expression of co-stimulatory surface molecules (CD86 and MHC II), M1-associated cytokines (CXCL10, CD86, MHC II, IL-12, IL-6 and TNF-α) and down-regulation of immunoregulatory molecules (Arg-1 and CD206).

In this study, the percentage of matured DCs in spleen and peripheral blood was not changed after cryo-thermal therapy with eosinophils depletion, while M1 macrophage was decreased. Meanwhile, eosinophils depletion resulted in a partial reduction of pro-inflammatory cytokines (IL-7, IL-10 and IL-6) in DCs, but a comprehensive down-regulation of M1-associated cytokines in macrophage, which indicated that cryo-thermal-activated eosinophils had a stronger effect on M1 macrophage polarization than DCs maturation. In our previous study, cryo-thermal therapy triggered M1 macrophage polarization on day 5 following the treatment, while fully maturation of DCs was emerged on day 14. Therefore, we suggested that M1 macrophage polarization occurred prior to DCs maturation after cryo-thermal therapy, and cryo-thermal-activated eosinophils firstly induced M1 macrophage polarization. However, the interactions of cryo-thermal-activated eosinophils, macrophages and DCs should be furtherly studied.

Cryo-thermal therapy induced the expression of IFN-γ and IL-4 in eosinophils from day 3 after the treatment. IFN-γ can enhance macrophage and DCs activation through TLR ligation^31^. IL-4 produced by eosinophils is always associated with anti-inflammatory activity and maintains the balance of Th1/Th2^45^. IL-4-expressing eosinophils play an important role in macrophage development^27,45^. In this study, blockade of IFN-γ produced by eosinophils inhibited DCs maturation, but IL-4 neutralization promoted DCs maturation, which suggested that cryo-thermal-activated eosinophils modulated DCs maturation via the antagonistic interaction between IFN-γ and IL-4. In addition, blockade of IFN-γ produced by eosinophils inhibited M1 macrophage polarization and promoted the activation of M2 macrophage. However, IL-4 neutralization down-regulated the expression of M1-associated cytokines and pleiotropic regulated the expression of immunoregulatory molecules. We speculated that, unlike the antagonistic effects of IFN-γ and IL-4 on DCs maturation, IFN-γ and IL-4 derived from cryo-thermal-activated eosinophils would synergistically promote M1 macrophage polarization. But the role of IFN-γ and IL-4 derived from cryo-thermal-activated eosinophils in M1 macrophage polarization and DCs maturation should be further investigated.

Effector T cells are the major players in adaptive immunity for tumour destruction. Activated CD8^+^ T cells have direct cytotoxic activity against tumour cells via the expression of cytotoxic perforin/granzyme-B and IFN-γ^46^. The optimal polarization of CD4^+^ T cells is essential to obtain durable systemic anti-tumour immunity^47^. CD4^+^ CTL and Th1 subsets mainly exert anti-tumour activity, while the regulatory T cells (Tregs) have potent tumour-promoting activity^48,49^. The function of other CD4^+^ T cell subsets, Th2 and Th17 depend on the certain circumstances^47^. Eosinophils have the ability to physically interact with CD4^+^ T cells, and provide costimulatory signals for the activation of CD4^+^ T cells^38^. In this study, Eosinophils depletion inhibited the differentiation of CD4^+^ T cells into CD4-CTL, and inhibited the differentiation of Tregs and Th17 cells. And low level of chronic exposure to Th17-associated cytokines may facilitate cancer progression^50^. The Th2-assoicated cytokine IL-4 was decreased, and IL-5 was up-regulated after eosinophils depletion. IL-4 produced by CD4^+^ T cells may inhibit angiogenesis, promote the infiltration of eosinophils and macrophages into tumour and exert anti-tumour effects, while IL-5 mainly has immunosuppressive and tumour-promoting function^47,51^. Generally, cryo-thermal-activated eosinophils could significantly promote the differentiation of CD4^+^ T cells into CD4-CTL subset and inhibit the differentiation of Tregs, Th17 and Th2 cells. Meanwhile, cryo-thermal-activated eosinophils could enhance the cytotoxic activity of CD8^+^ T cells. The result showed that cryo-thermal-activated eosinophils have the capacity to directly activate the anti-tumour CD4^+^ T cells and cytotoxic CD8^+^ T cells.

In this study, the expression of IL-10, inducible nitric oxide synthase (iNOS) and CCL22 in macrophages was decreased after eosinophils depletion. IL-10 acts as an anti-inflammatory cytokine which plays a key role in limiting the extent of immune activation^52^. The proper balance between IL-12 and IL-10 controls the appearance of immune response^53^. After eosinophils depletion, the decreased expression of IL-12 in macrophage would limit the expression of IL-10 to maintain the balance of IL-12/IL-10. iNOS is a critical pro-inflammatory cytokine which can mediate the production of nitric oxide (NO) and inhibit the production of macrophage-derived TGF-β^54^. NO is an essential mediator of macrophage mediated cytotoxicity in anti-tumour immunity^55^. CCL22 can contribute to the infiltration and inflammatory function of macrophage^56^. Hence, we speculated that the down-regulated expression of iNOS and CCL22 in macrophage after eosinophils depletion would hamper cryo-thermal-induced anti-tumour immunity.

In our study, the expression level of IDO1 was up-regulated after tumour-bearing DCs co-cultured with cryo-thermal-activated eosinophils as compared to tumour-bearing DCs co-cultured with tumour-bearing eosinophils. IFN-γ can induce the expression of IDO in DCs and IFN-γ-IDO axis is responsible for avoiding harmful hyperinflammatory response^57^. The high expression of IDO1 would play an important role in limiting exaggerated inflammatory reactions after cryo-thermal therapy. In addition, Arg-1 was expressed at a much higher level after tumour-bearing macrophage co-cultured with cryo-thermal-activated eosinophils as compared to tumour-bearing macrophage co-cultured with tumour-bearing eosinophils. The role of macrophage-derived Arg-1 in tumour immunity remains controversial^58^. Macrophage could induce anti-tumour activity through the depletion of L-arginine by Arg-1 within the tumour microenvironment^59^. So, the high level of Arg-1 in macrophage could be helpful for anti-tumour immunity after cryo-thermal therapy.

In our previous study, cryo-thermal therapy caused extensive tumor necrosis and elevated extracellular release of DAMPs (damage associated molecular patterns), such as HSP70, CRT and HMGB1 (unpublished data) from necrotic tumor cells^29^. Since DAMPs can act as a danger signal to activate eosinophils and prolong their lifespan^60,61^, we speculated that cryo-thermal therapy induced up-regulated DAMPs release would play a vital role in the activation of eosinophils. In the near future study, we will investigate the mechanism of cryo-thermal therapy induced activation of eosinophils based on the above hypothesis.

In summary, our study demonstrated that cryo-thermal therapy induced eosinophils activation at early stage after the treatment, which was a pivotal cell of the innate immune system involved in the regulation of cryo-thermal-induced adaptive immunity. Cryo-thermal-activated eosinophils could promote M1 macrophage polarization, and DCs maturation, meanwhile, cryo-thermal-activated eosinophils could directly promote CD4^+^ T cells differentiation into CD4^+^ CTL and generation of cytotoxic CD8^+^ T cells shaping strong systematic anti-tumour immune response, which enhanced the therapeutic efficacy for metastatic tumour.

## Materials and Methods

### Animal models

All animal experiments have been approved by the Animal Welfare Committee of Shanghai Jiao Tong University. Experimental methods were conducted according to the guidelines of Shanghai Jiao Tong University Animal Care (approved by Shanghai Jiao Tong University Scientific Ethics Committee). The female C57BL/6 mice were purchased directly from Shanghai Slaccas Experimental Animal Co., Ltd (China). Mice were maintained in isolated cages. At 6–8 weeks of age, the mice were injected subcutaneously in the right femoral region with 5 × 10^5^ B16F10 murine melanoma cells.

### Cryo-thermal therapy procedures

Cryo-thermal therapy was constructed as previously described in detail^20,29^. Briefly, the mice were divided into two groups: tumour-bearing group without any treatment and cryo-thermal group on day 12 after tumour inoculation. The mice in cryo-thermal group were injected intraperitoneally (i.p.) with 1.6% pentobarbital sodium (0.5 ml/100 g, Sigma-Aldrich) for anesthesia. The subcutaneous tumour was freezed with liquid nitrogen at −20 °C for 5 min, then rewarming to 6–8 °C at room temperature and heated with radiofrequency (RF) at 50 °C for 10 min. The therapy system was developed by our laboratory. All the procedures were conducted at aseptic condition.

### Flow cytometry analysis

The spleen and peripheral blood were collected after therapy. Single-cell suspension of peripheral blood cells and splenocytes were prepared after treatment with erythrocyte-lysing reagent to remove red blood cells. For cell surface staining, the cells were stained with fluorescence conjugated antibodies which could bind cell specific surface marker for at 4 °C 30 min. For intercellular staining, the cells were stimulated with Cell Activation Cocktail (with Brefeldin A, 20 ug/ml) for 4 hours, cells were incubated with anti-FcγR antibody, followed by surface staining with corresponding surface marker antibodies then fixed, permeabilized and incubated with antibodies binding specific intercellular marker. Data were acquired using BD FACS Aria II cytometer (BD Biosciences) and analyzed using FlowJo software. Fixation Buffer, Intracellular Staining Permeabilization Wash Buffer and Cell Activation Cocktail (with Brefeldin A) were purchased from Biolegend (San Diego, CA). Fluorochrome-conjugated monoclonal antibodies: CD3-FITC (clone 145-2C11), CD11b-FITC (clone M1/70), CD11c-FITC (clone N418), CD86-PE (clone GL-1), I-A/I-E-PerCP/Cy5.5 (clone M5/114.15.2), Gr-1-PE (clone R86-8C5), IL-4 (clone 11B11) (all from Biolegend). Siglec-F-BV421 (clone E50-2440), CD4-APC (clone RM4–5), IFN-γ (clone XMG1.2) (all from BD Biosciences). CD8-APC (clone 53.6.7), F4/80-APC (clone BM8) (all from Sungene Biotech).

### Isolation of DCs, macrophages, eosinophils, CD4^+^ and CD8^+^ T cells

Single-cell suspension of splenocytes were prepared to isolate DCs, macrophages, eosinophils, CD4^+^ and CD8^+^ T cells. DCs were isolated by DC isolation micro-bread kit (StemCell) in line with manufacturer’s instructions. DCs with a purity of >90% were used for experiments. For macrophages isolation, splenocytes were plated in DMEM supplemented with 10% FBS at 37 °C in a humidified 5% CO_2_ incubator for 1 h, then the supernatant fraction was poured off and the adherent splenocyte fraction was processed for CD68^+^ macrophages isolation by using EasySepTM PE positive selection kit (StemCell) in line with manufacturer’s instructions. Macrophages with a purity of >90% were used for experiments. Siglec-F^+^ eosinophils were isolated by using EasySepTM PE positive selection kit (StemCell) in line with manufacturer’s instructions. Eosinophils with a purity of >90% were used for experiments. CD4^+^ and CD8^+^ T cells were isolated by Easysep^TM^ CD4^+^ T cell negative selection kit and Easysep^TM^ CD8^+^ T cell negative selection kit (StemCell), respectively. CD4^+^ and CD8^+^ T cells with a purity of >90% were used for experiments.

### RNA isolation and real-time qPCR

TRIzol Reagent (TaKaRa) was used to isolated total freshly RNA from purified splenic DCs, macrophages, eosinophils, CD4^+^ and CD8^+^ T cells. The RNA purity of all samples was measured using the absorbance at 260/280 nm, which was above 1.9. PrimeScript RT reagent kit (TaKaRa) was used to made cDNA. Reaction system for quantitative real-time qPCR was made using SYBR Premix Ex Taq (TaKaRa) in 284-well plates, and performed on ABI 7900HT sequence detection system (Applied Biosystems). Data was analyzed using SDS software. The primer sequence of each gene used in our study was submitted in Supplementary Table S1. Delta–delta Ct method was used to determine the relative mRNA expression level of each gene. All experiments were conducted in triplicates.

### Depletion of Eosinophils *in vivo*

For the depletion of eosinophils, 15 μg anti Siglec-F (clone 238047; R&D Systems) was injected intraperitoneally on day 1 before cryo-thermal therapy and day 3, 7, 11 after cryo-thermal therapy. Control groups consisted of mice were injected intraperitoneally with isotype-matched Ab (R&D Systems).

### *In vitro* cell co-culture assay

The splenic Siglec-F^+^ eosinophils were isolated from tumour-bearing mice on day 15 after the tumour inoculation and cryo-thermal treated mice on day 3 after therapy as previously described. The splenic CD11c^+^ DCs, CD68^+^ macrophages, CD4^+^ and CD8^+^ were isolated from tumour-bearing mice on day 15 following tumour inoculation. The tumour-bearing DCs or macrophages were co-cultured with eosinophils from tumour-bearing mice at a ratio of 1:1, respectively. The co-cultured cells were plated in DMEM supplemented with 20% serum from tumor-bearing mice at 37 °C in a humidified 5% CO_2_ incubator for 24 h. Meanwhile, the tumour-bearing DCs or macrophages were also co-cultured with eosinophils from cryo-thermal mice at a ratio of 1:1, respectively. The co-cultured cells were plated in DMEM supplemented with 20% serum from tumor-bearing mice at 37 °C in a humidified 5% CO_2_ incubator for 24 h. Furthermore, the tumour-bearing T cells were co-cultured with eosinophils from tumour-bearing or cryo-thermal mice at a ratio of 5:1, respectively. The co-cultured cells were plated in DMEM supplemented with 20% serum from tumor-bearing mice or cryo-thermal mice at 37 °C in a humidified 5% CO_2_ incubator for 24 h. The tumour-bearing DCs, macrophages and T cells were considered as control. The co-cultured cells were analyzed by flow cytometry, and purified for RT-qPCR assay.

### Cytokine blockade

For evaluated the effect of IL-4 and IFN-γ derived from eosinophils on DCs and macrophages *in vitro*, we used neutralizing antibodies to IL-4 or IFN-γ (anti-mouse IL-4 (clone 11B11), anti-mouse IFN-γ (clone XMG1.2), 10ug/ml, and all from Sungene Biotech) and the isotype control antibody Rat IgG1 (clone HRPN, Sungene Biotech).

### Cytotoxicity assay

For evaluating the eosinophils mediated cytotoxicity against tumour cells after cryo-thermal therapy, a colorimetric cell-counting kit (CCK-8, Dojindo laboratories) assay was used according to the manufacturer’s instructions. Briefly, splenic eosinophils were isolated from tumour-bearing or cryo-thermal-treated mice. Eosinophils were co-cultured with B16F10 cells at the ratio of eosinophils: B16F10 cells = 1:1, 2:1, 4:1, 8:1 and 16:1 at 37 °C in 5% CO_2_ (The mixed cells were co-cultured in 96-well plate, 2 × 10^4^ B16F10 cells in each well). After incubation for 24 hours, the supernatants were poured off, and the adherent cells were survival B16F10 cells. Then 90ul fresh DMEM and 10ul CCK-8 solution were added to each well and additionally incubated for 2 hours. And a microplate reader was used to measure the absorbance at 450 nm. The B16F10 cells were used as control, and cell culture media alone was used as blank, respectively. The survival rate of B16F10 cells was calculated as [(experimental-blank)]/[(control-blank)] × 100%.

Similar to the above, a CCK-8 assay was used to evaluate the cytotoxicity against tumour cells mediated by CD4^+^ or CD8^+^ T cells after cryo-thermal therapy with eosinophils depletion. Briefly, splenic CD4^+^ or CD8^+^ T cells were isolated from tumour-bearing mice, cryo-thermal with isotype treated mice and cryo-thermal with Siglec-F mAb treated mice. CD4^+^ or CD8^+^ T cells were co-cultured with B16F10 cells at the ratio of CD4^+^/CD8^+^ T cells: B16F10 cells = 1:2, 1:1, 4:1 and 8:1 at 37 °C in 5% CO_2_ (The mixed cells were co-cultured in 96-well plate, 2 × 10^4^ B16F10 cells in each well). After incubation for 24 hours, the supernatants were poured off, and the adherent cells were survival B16F10 cells. Then 90ul fresh DMEM and 10ul CCK-8 solution were added to each well and additionally incubated for 2 hours. And a microplate reader was used to measure the absorbance at 450 nm. The B16F10 cells were used as control, and cell culture media alone was used as blank, respectively. The survival rate of B16F10 cells was calculated as [(experimental-blank)]/[(control-blank)] × 100%.

### Tumor challenge after eosinophils depletion

To investigate the effect of eosinophils on systemic immunologic memory response after cryo-thermal therapy, primary B16F10 melanoma tumor in C57BL/6 mice were treated with cryo-thermal therapy, and eosinophils were depleted according to previous description(n = 6 per group). The living mice were rechallenged with 1 × 10^5^ B16F10 melanoma cells (i.v.) 45 days after the therapy. Two weeks later the mice were sacrificed for lung tumor nodules counting.

### Statistical analysis

The student’s t-test and two-way ANOVA were used for statistical comparisons using Graph Pad Prism 6. Figures denoted statistical significance of ^*^p < 0.05, ^**^p < 0.01, ^***^p < 0.001. *P*-values < 0.05 was considered to be statistically significant.on

## Supplementary information

Supplementary_information

## Data Availability

All data generated or analysed during this study are included in this published article (and its Supplementary Information files).

## References

[1] Calandri M (2018). Ablation of colorectal liver metastasis: Interaction of ablation margins and RAS mutation profiling on local tumour progression-free survival. European radiology.

[2] Butros SR, DelCarmen MG, Uppot RN, Arellano RS (2014). Image-guided percutaneous thermal ablation of metastatic pelvic tumor from gynecologic malignancies. Obstet Gynecol.

[3] Shady W (2016). Percutaneous Radiofrequency Ablation of Colorectal Cancer Liver Metastases: Factors Affecting Outcomes–A 10-year Experience at a Single Center. Radiology.

[4] Toraya-Brown S, Fiering S (2014). Local tumour hyperthermia as immunotherapy for metastatic cancer. Int J Hyperthermia.

[5] Kolosnjaj-Tabi J, Marangon I, Nicolas-Boluda A, Silva AKA, Gazeau F (2017). Nanoparticle-based hyperthermia, a local treatment modulating the tumor extracellular matrix. Pharmacol Res.

[6] He XZ (2015). Cryo-ablation improves anti-tumor immunity through recovering tumor educated dendritic cells in tumor-draining lymph nodes. Drug Des Devel Ther.

[7] Sabel MS, Arora A, Su G, Chang AE (2006). Adoptive immunotherapy of breast cancer with lymph node cells primed by cryoablation of the primary tumor. Cryobiology.

[8] Dewhirst MW, Lee CT, Ashcraft KA (2016). The future of biology in driving the field of hyperthermia. Int J Hyperthermia.

[9] Mehta A, Oklu R, Sheth RA (2016). Thermal Ablative Therapies and Immune Checkpoint Modulation: Can Locoregional Approaches Effect a Systemic Response?. Gastroenterology research and practice.

[10] Dong J, Liu P, Xu LX (2009). Immunologic response induced by synergistic effect of alternating cooling and heating of breast cancer. Int J Hyperthermia.

[11] Shen Y, Liu P, Zhang A, Xu LX (2008). Study on tumor microvasculature damage induced by alternate cooling and heating. Ann Biomed Eng.

[12] Xue T (2016). Interleukin-6 Induced “Acute” Phenotypic Microenvironment Promotes Th1 Anti-Tumor Immunity in Cryo-Thermal Therapy Revealed By Shotgun and Parallel Reaction Monitoring Proteomics. Theranostics.

[13] He K, Liu P, Xu LX (2017). The cryo-thermal therapy eradicated melanoma in mice by eliciting CD4(+) T-cell-mediated antitumor memory immune response. Cell Death Dis.

[14] Pozzi LA, Maciaszek JW, Rock KL (2005). Both dendritic cells and macrophages can stimulate naive CD8 T cells *in vivo* to proliferate, develop effector function, and differentiate into memory cells. J Immunol.

[15] Corrales L, Matson V, Flood B, Spranger S, Gajewski TF (2017). Innate immune signaling and regulation in cancer immunotherapy. Cell research.

[16] Tran Janco JM, Lamichhane P, Karyampudi L, Knutson KL (2015). Tumor-infiltrating dendritic cells in cancer pathogenesis. J Immunol.

[17] Datta J (2014). Optimizing Dendritic Cell-Based Approaches for Cancer Immunotherapy. The Yale Journal of Biology and Medicine.

[18] Mantovani A, Marchesi F, Malesci A, Laghi L, Allavena P (2017). Tumour-associated macrophages as treatment targets in oncology. Nat Rev Clin Oncol.

[19] Chanmee T, Ontong P, Konno K, Itano N (2014). Tumor-associated macrophages as major players in the tumor microenvironment. Cancers (Basel).

[20] He, K., Jia, S., Lou, Y., Liu, P. & Xu, L. X. Cryo-thermal therapy induces macrophage polarization for durable anti-tumor immunity. *Cell Death & Disease* **10**10.1038/s41419-019-1459-7 (2019). 10.1038/s41419-019-1459-7 PMC6399266 30833570

[21] Yang BG, Seoh JY, Jang MH (2017). Regulatory Eosinophils in Inflammation and Metabolic Disorders. Immune Netw.

[22] Saraiva AL, Carneiro F (2018). New Insights Into the Role of Tissue Eosinophils in the Progression of Colorectal Cancer: A Literature Review. Acta medica portuguesa.

[23] McNeel DG (2014). A transient increase in eosinophils is associated with prolonged survival in men with metastatic castration-resistant prostate cancer who receive sipuleucel-T. Cancer immunology research.

[24] Jain M, Kasetty S, Khan S, Jain NK (2014). Tissue eosinophilia in head and neck squamous neoplasia: an update. Experimental oncology.

[25] Sakkal S, Miller S, Apostolopoulos V, Nurgali K (2016). Eosinophils in Cancer: Favourable or Unfavourable?. Current medicinal chemistry.

[26] Lotfi R, Lotze MT (2008). Eosinophils induce DC maturation, regulating immunity. J Leukoc Biol.

[27] Qiu Y (2014). Eosinophils and type 2 cytokine signaling in macrophages orchestrate development of functional beige fat. Cell.

[28] Carretero R (2015). Eosinophils orchestrate cancer rejection by normalizing tumor vessels and enhancing infiltration of CD8(+) T cells. Nat Immunol.

[29] Zhu J (2016). Cryo-thermal therapy elicits potent anti-tumor immunity by inducing extracellular Hsp70-dependent MDSC differentiation. Scientific reports.

[30] Zumwalt TJ, Arnold M, Goel A, Boland CR (2015). Active secretion of CXCL10 and CCL5 from colorectal cancer microenvironments associates with GranzymeB+ CD8+ T-cell infiltration. Oncotarget.

[31] Sheng KC, Day S, Wright MD, Stojanovska L, Apostolopoulos V (2013). Enhanced Dendritic Cell-Mediated Antigen-Specific CD4+ T Cell Responses: IFN-Gamma Aids TLR Stimulation. J Drug Deliv.

[32] Kim HS (2015). STAT1 deficiency redirects IFN signalling toward suppression of TLR response through a feedback activation of STAT3. Sci Rep.

[33] Borges da Silva H, Fonseca R, Alvarez JM, D’Imperio Lima MR (2015). IFN-gamma Priming Effects on the Maintenance of Effector Memory CD4(+) T Cells and on Phagocyte Function: Evidences from Infectious Diseases. J Immunol Res.

[34] Padigel UM (2007). Eosinophils act as antigen-presenting cells to induce immunity to Strongyloides stercoralis in mice. J Infect Dis.

[35] Liu, K., He, K., Xue, T., Liu, P. & Xu, L. X. The cryo-thermal therapy-induced IL-6-rich acute pro-inflammatory response promoted DCs phenotypic maturation as the prerequisite to CD4(+) T cell differentiation. *International Journal of Hyperthermia the Official Journal of European Society for Hyperthermic Oncology North American Hyperthermia Group*, 1 (2017). 10.1080/02656736.2017.1332394 28540834

[36] Simson L (2007). Regulation of Carcinogenesis by IL-5 and CCL11: A Potential Role for Eosinophils in Tumor Immune Surveillance. The Journal of Immunology.

[37] Legrand F (2010). Human eosinophils exert TNF-alpha and granzyme A-mediated tumoricidal activity toward colon carcinoma cells. J Immunol.

[38] Gatault S, Legrand F, Delbeke M, Loiseau S, Capron M (2012). Involvement of eosinophils in the anti-tumor response. Cancer Immunol Immunother.

[39] Dudek AM, Martin S, Garg AD, Agostinis P (2013). Immature, Semi-Mature, and Fully Mature Dendritic Cells: Toward a DC-Cancer Cells Interface That Augments Anticancer Immunity. Front Immunol.

[40] Motta JM, Rumjanek VM (2016). Sensitivity of Dendritic Cells to Microenvironment Signals. J Immunol Res.

[41] Demoulin S, Herfs M, Delvenne P, Hubert P (2013). Tumor microenvironment converts plasmacytoid dendritic cells into immunosuppressive/tolerogenic cells: insight into the molecular mechanisms. J Leukoc Biol.

[42] Walsh KP, Mills KH (2013). Dendritic cells and other innate determinants of T helper cell polarisation. Trends Immunol.

[43] Hussein MR (2006). Tumour-associated macrophages and melanoma tumourigenesis: integrating the complexity. International journal of experimental pathology.

[44] Mosser DM, Edwards JP (2008). Exploring the full spectrum of macrophage activation. Nat Rev Immunol.

[45] Pajarinen J (2015). Modulation of mouse macrophage polarization *in vitro* using IL-4 delivery by osmotic pumps. J Biomed Mater Res A.

[46] Caserta S, Borger JG, Zamoyska R (2012). Central and effector memory CD4 and CD8 T-cell responses to tumor-associated antigens. Critical reviews in immunology.

[47] Kim HJ, Cantor H (2014). CD4 T-cell subsets and tumor immunity: the helpful and the not-so-helpful. Cancer Immunol Res.

[48] Pardoll DM, Topalian SL (1998). The role of CD4+ T cell responses in antitumor immunity. Current opinion in immunology.

[49] Facciabene A, Motz GT, Coukos G (2012). T-regulatory cells: key players in tumor immune escape and angiogenesis. Cancer Res.

[50] Pan B (2015). Interleukin-17 promotes angiogenesis by stimulating VEGF production of cancer cells via the STAT3/GIV signaling pathway in non-small-cell lung cancer. Sci Rep.

[51] Tepper RI, Pattengale PK, Leder P (1989). Murine interleukin-4 displays potent anti-tumor activity *in vivo*. Cell.

[52] Ma Xiaojing, Yan Wenjun, Zheng Hua, Du Qinglin, Zhang Lixing, Ban Yi, Li Na, Wei Fang (2015). Regulation of IL-10 and IL-12 production and function in macrophages and dendritic cells. F1000Research.

[53] Stanilov NS (2012). Monocytes expression of IL-12 related and IL-10 genes in association with development of colorectal cancer. Mol Biol Rep.

[54] Bogdan C (1998). The multiplex function of nitric oxide in (auto)immunity. J Exp Med.

[55] Kwon SJ, Lee GT, Lee JH, Kim WJ, Kim IY (2009). Bone morphogenetic protein-6 induces the expression of inducible nitric oxide synthase in macrophages. Immunology.

[56] Dogan RN (2011). CCL22 regulates experimental autoimmune encephalomyelitis by controlling inflammatory macrophage accumulation and effector function. J Leukoc Biol.

[57] Pallotta MT (2011). Indoleamine 2,3-dioxygenase is a signaling protein in long-term tolerance by dendritic cells. Nat Immunol.

[58] Colegio OR (2014). Functional polarization of tumour-associated macrophages by tumour-derived lactic acid. Nature.

[59] Ellyard JI, Quah BJ, Simson L, Parish CR (2010). Alternatively activated macrophage possess antitumor cytotoxicity that is induced by IL-4 and mediated by arginase-1. Journal of immunotherapy (Hagerstown, Md.: 1997).

[60] Lotfi R (2009). Eosinophils oxidize damage-associated molecular pattern molecules derived from stressed cells. Journal of immunology (Baltimore, Md.: 1950).

[61] Lotfi R, Lee JJ, Lotze MT (2007). Eosinophilic granulocytes and damage-associated molecular pattern molecules (DAMPs): role in the inflammatory response within tumors. Journal of immunotherapy (Hagerstown, Md.: 1997).

