# Supplementary material for: A role of eosinophils in mediating the anti-tumour effect of cryo-thermal treatment
Source: Sci Rep. 2019 Sep 13;9:13214. doi: 10.1038/s41598-019-49734-5 (PMC6744470; doi:10.1038/s41598-019-49734-5)
Supplement: Supplementary file 1 — Supplementary_information [file 41598_2019_49734_MOESM1_ESM.docx]

Supplementary information

**A role of eosinophils in mediating the anti-tumour effect of cryo-thermal treatment**

Shengguo Jia**^1^**, Wentao Li**^2,3^**, Ping Liu**^1,^ ***, Lisa X. Xu**^1^**

**^1^**School of Biomedical Engineering and Med-X Research Institute,

Shanghai Jiao Tong University, Shanghai, P.R. China;

**^2^**Fudan University Shanghai Cancer Center, Shanghai, China;

**^3^**Department of Oncology, Shanghai Medical College, Fudan University,

Shanghai, China

Corresponding Author (*):

Ping Liu: pingliu@sjtu.edu.cn

*
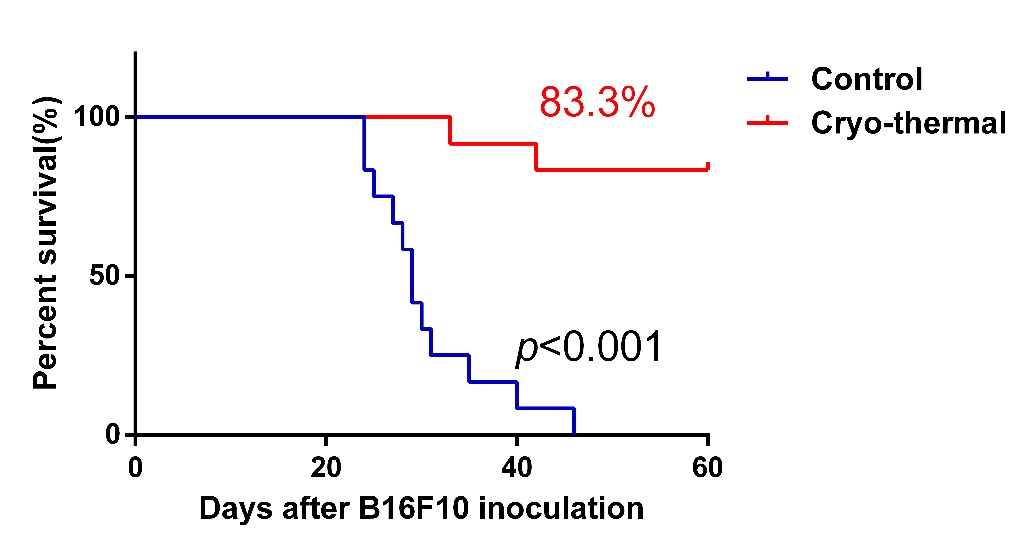
*

**Supplementary Figure 1** Kaplan–Meier survival curve was compared using log-rank tests. Survival rate of the cryo-thermal group was indicated; n=12 for control and the cryo-thermal group, respectively.





**Supplementary Figure 2** The phenotype changes of splenic DCs and macrophages on day 3 after cryo-thermal therapy. (**A**) Percentage of CD86^+^MHC II^+^ cells in splenic CD11c^+^DCs were analyzed by flow cytometry in cryo-thermal treated mice and untreated mice on day 3 after cryo-thermal therapy. (**B**) Percentage of CD86^+^MHC II^+^ cells in splenic CD11b^+^ F4/80^+^macrophages were analyzed by flow cytometry in cryo-thermal-treated mice and untreated mice on day 3 after cryo-thermal therapy. n=4 mice per group. Data was shown as mean ± SD.





**Supplementary Figure 3** The expression of IL-4 and IFN-γ in splenic eosinophils after cryo-thermal therapy. (**A**) The proportion of IL-4^+^ cells in splenic eosinophils in the cryo-thermal-treated mice or untreated mice was analyzed by flow cryometry. (**B**) The proportion of IFN-γ^+^ cells in splenic eosinophils in the cryo-thermal-treated mice or untreated mice was analyzed by flow cryometry. n=4 mice per group. Data was shown as mean ± SD. Data for bar graphs was calculated using student’s t-test. * p < 0.05; ** p <0.01; *** p < 0.001.





**Supplementary Figure 4** The changes of CD11c^+^DCs and F4/80^+^ macrophages after co-cultured with eosinophils. (**A**) Flow cryometry analysis of the CD11c^+^DCs after co-cultured with eosinophils. (**B**) Flow cryometry analysis of the F4/80^+^ macrophages after co-cultured with eosinophils. Data was shown as mean ± SD. Data for bar graphs was calculated using student’s t-test. ** p < 0.01.





**Supplementary Figure 5** Effect of cytotoxic against tumor cells mediated by CD4^+^ and CD8^+^ T cells after cryo-thermal therapy with eosinophils depletion. Splenic CD4^+^ and CD8^+^ T cells from tumor-bearing mice, cryo-thermal with isotype treated mice and cryo-thermal with Siglec-F mAb treated mice were purified and co-cultured with B16F10 cells at indicated ratios for 24h, and the cell viability (%) was determined by a CCK-8 assay. (**A**)The B16F10 cell viability (%) in co-cultured with tumor-bearing CD8^+^ T cells, cryo-thermal + isotype treated CD8^+^ T cells and cryo-thermal +Siglec-F mAb treated CD8^+^ T cells. (**B**) The B16F10 cell viability (%) in co-cultured with tumor-bearing CD4^+^ T cells, cryo-thermal + isotype treated CD4^+^ T cells and cryo-thermal +Siglec-F mAb treated CD4^+^ T cells. Data was shown as mean ± SD. Data for bar Data for bar graphs was calculated using student’s t-test. * p < 0.05; ** p < 0.01; *** p < 0.001 was considered to be statistically significant between cryo-thermal + Siglec-F mAb and cryo-thermal + isotype group.

**Supplementary Table S1.** Primers used for RT-PCR analysis

| **Gene** | **Primer** | **Sequence (5´-3´)** |
| --- | --- | --- |
| IFN-γ | forward  reverse | ATGAACGCTACACACTGCATC  CCATCCTTTTGCCAGTTCCTC |
| IL-6 | forward  reverse | GACAAAGCCAGAGTCCTTCAGAGAGATACAG  TTGGATGGTCTTGGTCCTTAGCCAC |
| IL-12 | forward  reverse | TGGTTTGCCATCGTTTTGCTG  ACAGGTGAGGTTCACTGTTTCT |
| IL-15 | forward  reverse | AGAGGCCAACTGGATAGATGT  AGAGCACGTTTCTTACTGTTTCA |
| TNF-α | forward  reverse | TTCTGTCTACTGAACTTCGGGGTGATCGGTCC  GTATGAGATAGCAAATCGGCTGACGGTGTGGG |
| CCL5 | forward  reverse | GCTGCTTTGCCTACCTCTCC  TCGAGTGACAAACACGACTGC |
| CXCL10 | forward  reverse | CCAAGTGCTGCCGTCATTTTC  GGCTCGCAGGGATGATTTCAA |
| MHC II | forward  reverse | AGCCCCATCACTGTGGAGT  GATGCCGCTCAACATCTTGC |
| CD86 | forward  reverse | GAGCTGGTAGTATTTTGGCAGG  GGCCCAGGTACTTGGCATT |
| Perforin | forward  reverse | CTGCCACTCGGTCAGAATG  CGGAGGGTAGTCACATCCAT |
| Granzyme-B | forward  reverse | CCACTCTCGACCCTACATGG  GGCCCCCAAAGTGACATTTATT |
| IL-4 | forward  reverse | CCCCAGCTAGTTGTCATCCTG  CAAGTGATTTTTGTCGCATCCG |
| IL-1β | forward  reverse | ACAGCAGCACATCAACAAGAG  ATGGGAACGTCACACACCAG |
| IL-7 | forward  reverse | TTCCTCCACTGATCCTTGTTCT  AGCAGCTTCCTTTGTATCATCAC |
| IL-10 | forward  reverse | GCTCTTACTGACTGGCATGAG  CGCAGCTCTAGGAGCATGTG |
| HO-1 | forward  reverse | AAGCCGAGAATGCTGAGTTCA  GCCGTGTAGATATGGTACAAGGA |
| STAT3 | forward  reverse | AATATAGCCGATTCCTGCAAGAG  TGGCTTCTCAAGATACCTGCTC |
| Foxo3 | forward  reverse | CTGGGGGAACCTGTCCTATG  TCATTCTGAACGCGCATGAAG |
| IDO2 | forward  reverse | CCAGAAGGACCGTTGGAAATC  ACTGTCACTAGGATGAAGCCC |
| IDO1 | forward  reverse | CAAAGCAATCCCCACTGTATCC  ACAAAGTCACGCATCCTCTTAAA |
| VEGFR2 | forward  reverse | TTTGGCAAATACAACCCTTCAGA  GCAGAAGATACTGTCACCACC |
| PD-L1 | forward  reverse | GCTTCTCAATGTGACCAGCA  GAGGAGGACCGTGGACACTA |
| iNOS | forward  reverse | ACATCGACCCGTCCACAGTAT  CAGAGGGGTAGGCTTGTCTC |
| Arg-1 | forward  reverse | TTGGGTGGATGCTCACACTG  GTACACGATGTCTTTGGCAGA |
| CD206 | forward  reverse | GCAGGTGGTTTATGGGATGT  GGGTTCAGGAGTTGTTGTGG |
| Eomes | forward  reverse | GGCCCCTATGGCTCAAATTCC  CCTGCCCTGTTTGGTGATG |
| IL-2 | forward  reverse | TGAGCAGGATGGAGAATTACAGG  GTCCAAGTTCATCTTCTAGGCAC |
| T-bet | forward  reverse | GTTCAACCAGCACCAGACAGAG  TGGTCCACCAAGACCACATC |
| CCL20 | forward  reverse | TACAGACGCCTCTTCCTTCCA  CAGCCCTTTTCACCCAGTTC |
| IL-17A | forward  reverse | GAAGGCCCTCAGACTACCTCAA  TCATGTGGTGGTCCAGCTTTC |
| RORγt | forward  reverse | CGCGGAGCAGACACACTTA  CCCTGGACCTCTGTTTTGGC |
| TGF-β | forward  reverse | CTCCCGTGGCTTCTAGTGC  GCCTTAGTTTGGACAGGATCTG |
| Foxp3 | forward  reverse | AGCAGTGTGGACCGTAGATGA  GGCAGGGATTGGAGCACTT |
| IL-13 | forward  reverse | CGGCAGCATGGTATGGAGTG  ATTGCAATTGGAGATGTTGGTCAG |
| IL-5 | forward  reverse | TCAGGGGCTAGACATACTGAAG  CCAAGGAACTCTTGCAGGTAAT |
| GATA3 | forward  reverse | GGATGTAAGTCGAGGCCCAAG  ATTGCAAAGGTAGTGCCCGGTA |
| GAPDH | forward  reverse | AGGTCGGTGTGAACGGATTTG  GGGGTCGTTGATGGCAACA |
